# Multi-dimensional predictors of first drinking initiation and regular drinking onset in adolescence: A prospective longitudinal study

**DOI:** 10.1016/j.dcn.2024.101424

**Published:** 2024-07-30

**Authors:** Tam T. Nguyen-Louie, Wesley K. Thompson, Edith V. Sullivan, Adolf Pfefferbaum, Camila Gonzalez, Sonja C. Eberson-Shumate, Natasha E. Wade, Duncan B. Clark, Bonnie J. Nagel, Fiona C. Baker, Beatriz Luna, Kate B. Nooner, Massimiliano de Zambotti, David B. Goldston, Brian Knutson, Kilian M. Pohl, Susan F. Tapert

**Affiliations:** a Department of Psychiatry, University of California, San Diego, CA, USA; b Center for Population Neuroscience and Genetics, Laureate Institute for Brain Institute, Tulsa, OK, USA; c Department of Psychiatry and Behavioral Sciences, Stanford University School of Medicine, Stanford, CA, USA; d Center for Health Sciences, SRI International, Menlo Park, CA, USA; e Department of Psychiatry, University of Pittsburgh, Pittsburgh, PA, USA; f Departments of Psychiatry and Behavioral Neuroscience, Oregon Health and Science University, Portland, OR, USA; g School of Physiology, University of the Witwatersrand, Johannesburg, South Africa; h Department of Psychology, University of North Carolina Wilmington, Wilmington, NC, USA; i Department of Psychiatry and Behavioral Sciences, Duke University School of Medicine, Durham, NC, USA; j Department of Psychology, Stanford University, Stanford, CA 94305, USA

**Keywords:** Adolescent alcohol use onset, Regular drinking onset, Time-to-event models, NCANDA, Withdrawal, Binge drinking

## Abstract

Early adolescent drinking onset is linked to myriad negative consequences. Using the National Consortium on Alcohol and NeuroDevelopment in Adolescence (NCANDA) baseline to year 8 data, this study (1) leveraged best subsets selection and Cox Proportional Hazards regressions to identify the most robust predictors of adolescent first and regular drinking onset, and (2) examined the clinical utility of drinking onset in forecasting later binge drinking and withdrawal effects. Baseline predictors included youth psychodevelopmental characteristics, cognition, brain structure, family, peer, and neighborhood domains. Participants (N=538) were alcohol-naïve at baseline. The strongest predictors of first and regular drinking onset were positive alcohol expectancies (Hazard Ratios [HRs]=1.67–1.87), easy home alcohol access (HRs=1.62–1.67), more parental solicitation (e.g., inquiring about activities; HRs=1.72–1.76), and less parental control and knowledge (HRs=.72–.73). Robust linear regressions showed earlier first and regular drinking onset predicted earlier transition into binge and regular binge drinking (βs=0.57–0.95). Zero-inflated Poisson regressions revealed that delayed first and regular drinking increased the likelihood (Incidence Rate Ratios [IRR]=1.62 and IRR=1.29, respectively) of never experiencing withdrawal. Findings identified behavioral and environmental factors predicting temporal paths to youthful drinking, dissociated first from regular drinking initiation, and revealed adverse sequelae of younger drinking initiation, supporting efforts to delay drinking onset.

## Highlights

• Pre-drinking youth, family, peer, and neighborhood characteristics all predict alcohol onset.
• Youth personality/disposition and parental involvement are most predictive of alcohol use onset.
• Age at first drink is a stronger predictor of withdrawal symptoms than weekly drinking onset age.
• Delaying first and weekly drinking onset may mitigate potential downstream deleterious effects.

## 1 Introduction

Alcohol, although illegal for purchase and consumption under age 21 years in the United States, is the most commonly used substance among US youth (Miech et al., 2023).An earlier age of first alcohol use is associated with myriad deleterious outcomes, including alcohol-related legal and vocational consequences (Gruber et al., 1996), greater binge drinking frequency (Morean et al., 2014), alcohol use disorder (AUD; Dawson et al., 2008; DeWit et al., 2000; Hingson et al., 2006), and other substance use (Pilatti et al., 2013). A potentially more robust predictor of these alcohol-related outcomes is age of regular, or habitual (e.g., weekly), drinking onset rather than age at first drink (Kuntsche et al., 2013, Warner and White, 2003). Among 1500 adolescents 13 – 19 year-olds, both age of first and regular drinking onset predicted subsequent AUD, but the relationship was stronger for the latter (Sartor et al., 2016).

The well-recognized association between alcohol use onset age and subsequent problematic drinking highlights the potential utility of delaying initiation to reduce AUD and concomitant deleterious outcomes (Connor et al., 2019, Grant and Dawson, 1997, Guttmannova et al., 2012). While many studies have examined risk factors of problematic adolescent alcohol *use*, few have focused on pre-drinking factors that forecast adolescent drinking *onset*. The available evidence suggests some overlap between earlier ages of drinking onset and riskier adolescent drinking patterns, as heralded by externalizing behaviors, being male sex (versus female), lower parental control, higher parent substance use (Maggs et al., 2019), lower parent education (Visser et al., 2015), positive AUD family history (Dawson et al., 2008), and higher peer use (Armenta et al., 2016). Despite this overlap, drinking onset and problematic drinking behaviors are distinct phenomena, such that not all youth who initiate drinking early will transition into risky drinking. Identifying unique predictors of drinking onset compared with regular drinking may offer important preventative targets before alcohol use escalates into chronic use among youth.

Following an ecological framework, predictors of adolescent alcohol use can be organized based on levels of influence (Trucco, 2020). One such framework, the Bioecological Model (Bronfenbrenner, 1974), posits that development is affected by increasingly more complex interactions between individuals and their immediate environment. At the lowest level, the interplay between the environment and person varies based on individual psychodevelopmental characteristics (Bronfenbrenner and Ceci, 1994). For example, the most robust individual-level predictors of alcohol use include cortical thickness in frontal and limbic regions (Brumback et al., 2016, Rane et al., 2022), cognitive functioning (Squeglia et al., 2017), academic achievement (Maggs et al., 2008), and internalizing/externalizing symptomology (Farmer et al., 2016, Meque et al., 2019). The next level consists of the immediate family context, including parental involvement such as parental solicitation, or the extent to which parents inquire about youth activities and behaviors (Bray et al., 2022, Fletcher et al., 2004) and family history of AUD (Warner and White, 2003). The next levels are peer influences (e.g., peer use; Leung et al., 2014) and the neighborhood (e.g., alcohol outlet density; Morrison et al., 2019). At the broadest level of the Bioecological Model are cultural and sociopolitical influences shaping behaviors (e.g., norms and laws).

**Fig. 1:**
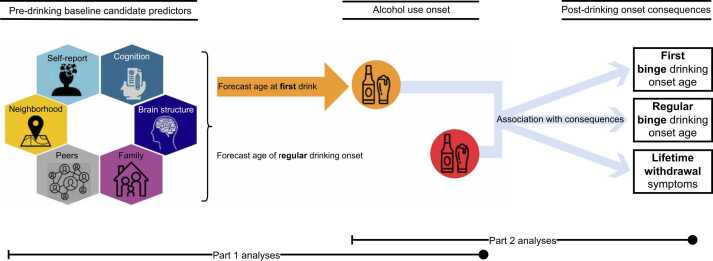
Study design summary. In a two-part analysis, this study used automated variable selection (best subset selection) to identify the subset of baseline predictors that best forecasted age at first drink and age of regular drinking onset. In part 2, age of first and regular drinking onset were then used to forecast subsequent transition into binge drinking, regular binge drinking, and lifetime withdrawal symptoms.

Using this framework, the current study aimed to identify significant pre-drinking initial (i.e., at study entry) predictors of alcohol use onset and characterize subsequent outcomes of drinking onset in a two-part analysis (Fig. 1).

Two metrics of drinking onset were examined: age at first drink (First Drinking Onset) and age of regular, or weekly, drinking onset (Regular Drinking Onset). While positively correlated, these metrics are not synonymous, as not all youth who initiate drinking will transition into regular use. First and Regular Drinking Onset were also examined simultaneously to ascertain the dissociative utility of each in predicting future drinking behaviors.

Part 1 (*Best subset of baseline precursors to alcohol use onset*) leveraged best subsets selection (Bertsimas et al., 2016), an automated variable selection method, and Cox Proportional Hazard (PH) regressions to identify the most robust baseline predictors (i.e., independent variables) in forecasting drinking onset among 100 candidate precursors. Pre-drinking initial predictors were derived from assessments of youth psychodevelopmental characteristics (e.g., personality traits, sleep patterns), cognition (e.g., executive functioning), brain structure (e.g., parahippocampal surface area), family (e.g., parental solicitation), peer (e.g., dating history), and neighborhood (e.g., median household income by ZIP code) domains. It was hypothesized that the strongest baseline predictors of problematic adolescent alcohol use identified in previous studies would also emerge as the strongest baseline predictors of earlier First and Regular Drinking Onset ages: low academic achievement, internalizing/externalizing traits, small frontal and limbic brain volumes, poor executive functioning, positive family history of alcohol problems, low parental involvement with youth, peer use, and high neighborhood alcohol outlet density.

Part 2 (*Prospective consequences of early alcohol use onset*) characterized the associations between each onset measure (First and Regular) and future binge drinking and alcohol withdrawal effects, as earlier onset has been associated with greater binge drinking frequency (Hingson and Zha, 2009) and AUD symptomology (Dawson et al., 2008, Grant and Dawson, 1997). A low prevalence of AUD was expected among the present sample of adolescents; instead, analyses focused on drinking behaviors that may serve as risk factors for development of AUD, notably intense drinking associated with adverse physiological sequelae (i.e., withdrawal symptoms; Martin and Winters, 1998). Both earlier First and Regular Drinking Onset were expected to forecast earlier binge drinking onset and more withdrawal effects. Regular Drinking Onset was expected to show a stronger relationship with binge drinking onset and withdrawal symptoms than First Drinking Onset. Together, this two-part analysis will provide a comprehensive view of adolescent drinking initiation, baseline predictors thereof, and clinically relevant prospective outcomes of early adolescent alcohol use onset.

## 2 Material and methods

### 2.1 Participants

Data were drawn from the ongoing National Consortium on Alcohol and NeuroDevelopment in Adolescence (NCANDA) study (Baseline to Year 8 Data Release of National Institute of Mental Health Data Archive Collection C4513). Participants were recruited and followed at Oregon Health & Science University (OHSU), SRI International, University of California, San Diego (UCSD), University of Pittsburgh Medical Center (UPMC), and Duke University Medical Center (DUMC) (Brown et al., 2015). Following a cohort sequential design, 831 youth were recruited in 2012 – 2014 in three age bands (12–14 years, 15–17 years, and 18–21 years).

**Fig. 2:**
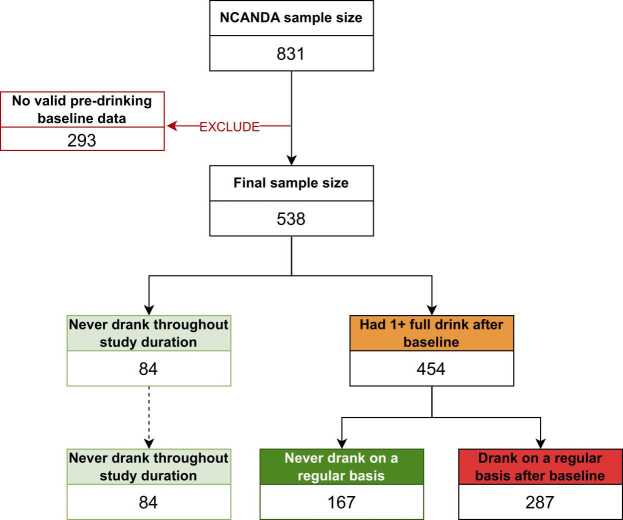
Sample selection criteria. Data for the present study were drawn from the National Consortium on Alcohol and NeuroDevelopment (NCANDA) study (N = 831); 293 participants were excluded due to initiating drinking prior to study entry, structural brain anomalies, or other missing baseline data. The final sample size consisted of 538 participants with valid pre-drinking baseline data. *Note:* Drinking on a regular basis indicates consuming ≥1 standard drink at least once a week for ≥3 consecutive months.

Exclusion criteria for NCANDA were age younger than 12 or older than 21 years at study entry, limited English fluency, MRI contraindications, current psychotropic medication use, non-correctable sensory problems, history of serious medical conditions that may affect MRI, early developmental problems (e.g., prenatal alcohol or illicit drug exposure); persistent Axis I mental health disorder, head trauma or loss of consciousness (>2 min), and severe learning or other pervasive developmental disorder. The current study further excluded participants (Fig. 2) who had at least one full drink at or prior to study entry (n = 265) as prospective examinations of drinking onset predictors were not possible among individuals who had already transitioned, participants who were missing pre-drinking self-report survey data or had data only at baseline (n = 14), or youth who were found to have structural brain anomalies precluding automated quantification (n = 14). In total, the current study consisted of 538 participants.

Informed consent was obtained from adult participants and parents/legal guardians for minor participants under 18 years-old, who provided written assent. Study protocol and procedures were approved by the Institutional Review Board of each study site. Participants were followed annually with clinical assessment of substance use, neuropsychological performance, mental health, and neuroimaging. Youth and parents/guardians were administered self-reports assessing major life domains (e.g., sociodemographic, peer relations, parental relations). Parents/guardians reported on parental education and family history of alcohol use problems. Data in the present study were collected at baseline through the 8-year follow-up.

Participants included in the present analyses (N = 538) were younger at study entry (*M* = 15.3, *SD* = 2.2 years old) than those excluded from analyses (N = 293; *M* = 17.9, *SD* = 2.2 years old; *t*_612_ = 16.4, *p* <.0001). Among drinkers, included participants transitioned into first drinking at age 17.8 (*SD* = 2.1 years), later than excluded participants (15.9 ± 2.2 years, *t*_602_ = 12.1, *p* <.0001). Included (*M* = 19.5, *SD* = 1.8) versus excluded participants (M = 19.2, SD = 2.4) did not differ in age of regular drinking onset. When examined by recruitment age band, the present sample included 91.7 % of youth recruited at 12 – 14, 61.2 % of youth recruited at 15 – 17, and 33.7 % of youth recruited at 18 – 21, a statistically significant difference in proportions among included versus excluded participants by recruitment age band, χ^2^ (2, 831) = 187.6, p <.0001. There were no differences in sex or other sociodemographics variables among included versus excluded participants.

Of the 538 youth included in the analyses, substance use was minimal at study entry, with participants naïve to lifetime use of tobacco (97.6 %), marijuana (96.5 %), and other drugs (99.8 %). Over the next 8 years, 454 (84.4 %) had at least one standard drink and 84 (15.6 %) remained alcohol-naïve. Among the 454 who initiated drinking, 287 transitioned into regular (i.e., weekly) drinking, while 167 remained non-regular (i.e., infrequent) drinkers. Among drinkers, 367 had transitioned into binge drinking, and 114 into regular binge drinking across the period of analysis; 174 had experienced at least one withdrawal symptom through their lifetime (see Table 1, Table 2 and Fig. 2). Distributions of the time between first to regular drinking onset and binge to regular binge drinking onset are shown in the supplement.

**Table 1:** Participant baseline characteristics at study entry (N = 538).

	*M* (*SD*) [Range] or *n* (%)
Age at study entry	15.3 (2.2) [12.0–21.4]
Sex at birth (female)	273 (50.7 %)
White	381 (70.8 %)
Hispanic	57 (10.6 %)
Family history density of alcohol use problems^a^	0.40 (0.69) [0.0 – 3.0]
Parent educational attainment (years)	16.3 (2.4)

a Calculated based on first- (parents) and second-(grandparents) degree relatives who experienced two or more alcohol-related consequences (Rice et al., 1995).

**Table 2:** Follow-up alcohol use transition characteristics (N = 538).

	*N (%)*	*M* (*SD*) [Range]
Age of first drinking onset	454 (84 %)	17.8 (2.1) [12.5 – 26.3]
Age of regular drinking onset	287 (53 %)	19.5 (1.8) [13.5 – 25.3]
Age of first binge drinking onset	367 (68 %)	18.6 2.1) [13.5 – 27.0]
Age of regular binge drinking onset	114 (21 %)	19.1 (1.7) [13.5 – 24.8]
Number of lifetime withdrawal symptoms	174 (32 %)	4.0 (3.6) [1.0 – 21.0]

*Note:* Ages are in years.

**Age of first drinking onset:** age at which youth first consumed 1+ standard drink in one sitting. **Age of regular drinking onset**: age at which youth first consumed 1+ standard drink at least once per week for 3+ consecutive months. **Age of first binge drinking onset**: age in which youth first consumed 4+ standard drinks (females) or 5+ standard drinks (males) in one sitting. **Age of regular binge drinking onset**: age at which youth first reported binge drinking at least once a week for 3+ consecutive months. **Withdrawal symptoms**: number of post-drinking symptoms youth reported experiencing from first drinking onset through Year 8 follow-up.

### 2.2 Measures

#### 2.2.1 Alcohol use variables

The Customary Drinking and Drug Use Record (CDDR; Brown et al., 1998) assesses use patterns, severity, and substance use disorder criteria for alcohol, nicotine, cannabis, and other drugs. Variables of interest were First Drinking Onset, Regular Drinking Onset, age of first binge drinking onset, age of regular binge drinking onset, and number of lifetime withdrawal symptoms. All variables were examined continuously. First Drinking Onset and Regular Drinking Onset acted as the outcomes of interest in Part 1 (*Best subset of baseline precursors to alcohol use onset*) and were in turn examined as the primary predictors (i.e., independent variables) of interest in Part 2 (*Prospective consequences of early alcohol use onset*). Age of binge and regular binge drinking onset, and lifetime withdrawal symptoms were selected as outcomes of interest in Part 2 analyses.

First Drinking Onset was defined as the age at which youth first consumed ≥1 standard drink. Regular Drinking Onset indicated the age at which youth first consumed ≥1 standard drink at least once a week for ≥3 consecutive months (Brown et al., 1998). Age of first binge drinking onset was defined as the age in which youth first consumed ≥4 standard drinks (females) or ≥5 standard drinks (males) in a single setting. Age of regular binge drinking onset indicated the age at which youth first reported binge drinking at least once a week for ≥3 consecutive months (Brown et al., 1998).

Withdrawal symptoms were defined as experiencing alcohol-related effects (e.g., shaking, sweating/rapid breathing, irritability, increased nervousness, insomnia) within two days of stopping or decreasing alcohol use. The most common symptoms reported in the current sample (N = 538) were stomach upset, nausea, vomiting, headaches, unclear or fuzzy thinking, and feeling weak or faint upon sitting down or standing up. All other alcohol withdrawal symptoms queried in the CDDR were reported at least once, except for auditory hallucinations, which were not experienced by any youth in the sample. Lifetime withdrawal symptoms were calculated as the cumulative number of withdrawal symptoms youth experienced from first drinking onset up to the most current year of NCANDA data release (i.e., Year 8).

#### 2.2.2 Drinking onset predictors of interest

All model predictors were time invariant and assessed at the first assessment upon study entry (i.e., baseline) for temporal capture of characteristics prior to alcohol use initiation. These initial baseline predictors of First and Regular Drinking Onset were organized into 6 domains consistent with the Bioecological Model (Bronfenbrenner and Ceci, 1994): youth psychodevelopmental characteristics, cognition, brain structure, family, peer, and neighborhood factors. Variables associated with these domains, other than brain structure, are described in further detail in Table 3 and the supplement.

**Table 3:** Non-MRI baseline predictors of first and regular drinking onset.

**Metric of interest**	**Measure**	**Source**
**Youth level psychodevelopmental characteristics**		
Sex	Clinical interview	Brown et al. (2015)
Pubertal development	Pubertal Developmental Scale	Carskadon and Acebo (1993); Peterson et al. (1988)
Academic functioning	Grade point average; future career intentions	Brown et al. (2015)
Personality/traits	Ten Item Personality Inventory (TIPI)^a^	Gosling et al. (2003)
	Urgency-Premeditation-Perseverance-Sensation Seeking-Positive Urgency (UPPS-P) Impulsive Behavior Scale^b^	Cyders et al. (2007); Lynam et al. (2006)
	Behavior Rating Inventory of Executive Function – Self-Report Version (BRIEF-SR)^c^	Gioia et al. (2002)
	Alcohol Expectancies Questionnaire^d^	Brown et al. (1987)
	Youth Self-Report (£17 years) and Adult Self-report (³18 years) Internalizing and Externalizing subscales	Achenbach (1991); Achenbach and Rescorla (2003)
Sleep patterns	Cleveland Adolescent Sleepiness Questionnaire; Composite Scale of Morningness	Smith et al. (2021); Spilsbury et al. (2007)
**Cognition**^**e**^		
Working memory	Penn Continuous Performance Test-Number Letter Version	All Penn Computerized Neurocognitive Battery tasks have been previously described by Gur et al. (2010).
Visual learning and memory	Penn Short Visual Object Learning Test (immediate and delayed)	
	Penn Facial Memory Test (immediate and delayed)	
	Penn Word Memory Test (immediate and delayed)	
Executive functioning	Penn Conditional Exclusion Task	
	Penn Matrix Analysis Test	
	Penn Logical Reasoning Task	
Affect processing	Penn Measured Emotion Differentiation Task	
	Penn Emotion Recognition Test	
**Family factors**		
Socioeconomic status	Race; ethnicity; parental educational attainment; parental marital status; current living arrangement	Brown et al. (2015)
Family history	Family history density of alcohol related problems	Rice et al. (1995)
Perceived access to alcohol	Access to Substances & Neighborhood Strength questionnaire	Komro et al. (2007); Tobler et al. (2009)
Youth-parent relations	Parental warmth, solicitation, knowledge, control, and supervision	Fletcher et al. (2004); Loeber et al. (1998); Sartor et al. (2016)
**Peer factors**		
Social network	Number of same sex friends; number of opposite sex friends	Brown et al. (2015)
Peer influence	Number of friends who drink alcohol, get drunk, or have problems with alcohol	Bachman (1981)
Romantic relationships	Dating history	Brown et al. (2015)
**Neighborhood factors**		
Population-level socioeconomic factors	ZIP code-based population metrics on median household income, poverty, educational attainment, public assistance recipients, and unemployment	American Community Survey (U.S. Census Bureau, 2022)
Alcohol outlet density	ZIP code-based quantity of alcohol-related establishments (beer/wine/liquor stores, bars)	County Business Patterns (U.S. Census Bureau, 2023a)

^a^TIPI subscales examined: Agreeableness, Conscientiousness, Emotional Stability, Extraversion, and Openness to Experiences; ^b^ UPPS subscales examined: Negative Urgency, Lack of Premeditation, Lack of Perseverance, Positive Urgency, and Sensation Seeking; ^c^ BRIEF-SR scales examined: Inhibitory Control, Flexibility, Emotional Control, Monitoring, Working Memory, Planning, Organization, and Task-Completion; ^d^ AEQ scales examined: Changes in Social Behavior, Increased Arousal, Improved Cognitive and Motor Ability, Relaxation and Tension Reduction, and Global Positive Change; ^e^Neuropsychological functioning was assessed using the Penn Computerized Neurocognitive Battery.

Regarding brain structure, the predictors of interest were based on the outcome of the Scalable Informatics for Biomedical Imaging Studies (SIBIS) processing pipeline, as described by Pfefferbaum (2018), applied to the baseline T1- and T2-weighted 3D structural Magnetic Resonance Images (MRI) acquired on Siemens 3 T TIM TRIO scanners (at sites UPMC and OHSU) and on General Electric 3 T Discovery MR750 scanners (at sites UCSD, SRI International, and DUMC). See Pfefferbaum et al., 2018, Pfefferbaum et al., 2016 for further details. For each MRI, the pipeline extracted FreeSurfer scores of 34 cortical Regions of Interest (ROI) defined by the Desikan-Killiany Atlas and 30 other brain structures (subcortical, ventricles, cerebellum, white matter hyperintensities, intracranial volume). Intracranial volume and scanner type (i.e., GE or Siemens) were regressed out from the ROI measurements to minimize head size differences associated with sex and ethnicity (Pfefferbaum et al., 2016) or scanner differences.

### 2.3 Candidate predictor selection

For all domains, candidate initial predictors were selected in a theory-driven approach based on characteristics identified in prior research as significantly associated with adolescent alcohol use (for reviews, see Hill and O’Brien, 2015; Squeglia and Cservenka, 2017). A search of the academic literature (i.e., published primary research articles, and review papers) and book chapters was conducted using PubMed, Web of Science, and Google Scholar, commenced September 8, 2023 and concluded December 4, 2023. Given the array of potential predictors of interest, search terms were broad (e.g., “adolescent drinking onset”) and pertained to adolescent alcohol use, age of onset, and longitudinal studies. Reference lists of each selected article and book chapter were manually reviewed to identify additional studies and guide subsequent search criteria.

**Fig. 3:**
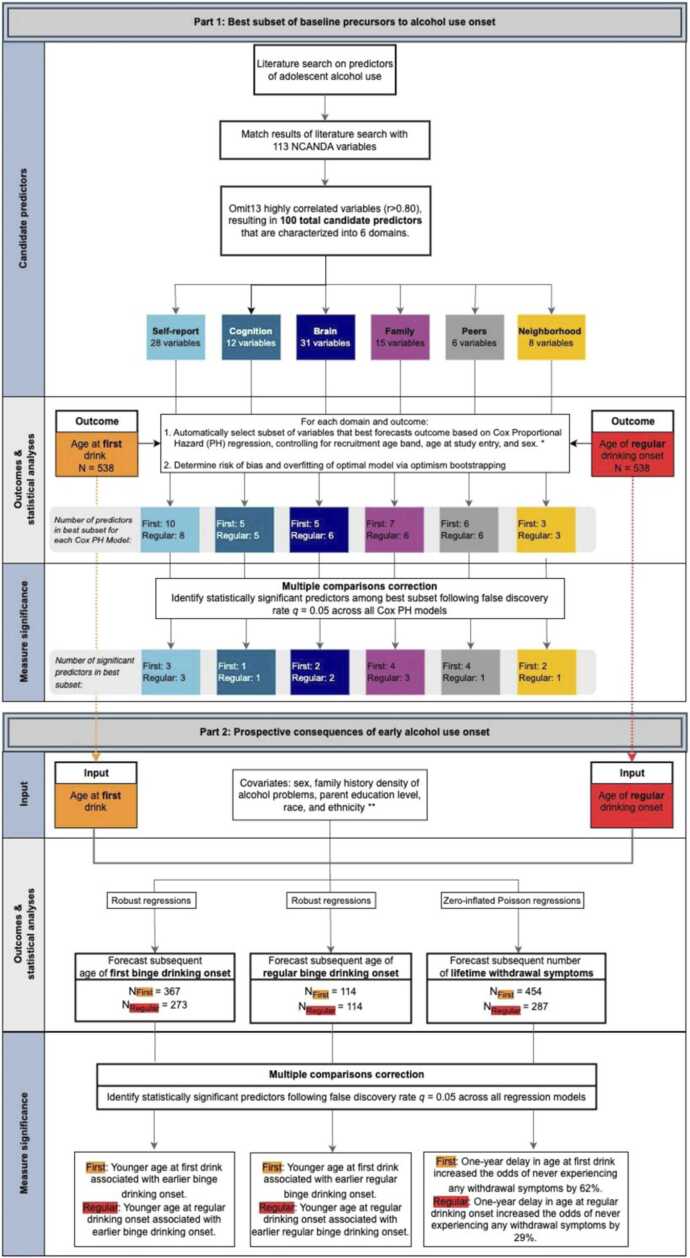
Study methods and statistical analyses flowchart. The present study was conducted in a two-part analysis. Part 1 utilized automated variable selection (best subsets selection) to select the most robust baseline predictors of adolescent first and regular drinking onset among 100 total candidate predictors in time-to-event Cox Proportional Hazards regressions. Part 2 employed robust regressions to longitudinally model linear outcomes (age of first and regular binge drinking onset) and zero-inflated Poisson regressions to model count outcomes with excessive zeros (number of lifetime withdrawal symptoms). * Cox Proportional Hazard models for the brain structure domain also accounted for head size and scanner type. ** Zero-inflated Poisson regressions also accounted for age at study entry. *Note*: N_First_ indicates sample sizes for models in which age of first drinking onset served as the primary predictor; N_Regular_ indicates sample sizes for models examining age of regular drinking onset as the primary predictor.

With respect to brain structure, a recent systematic review of neuroanatomical predictors of adolescent drinking (Honarvar et al., 2023) that commenced January 6, 2023 served as the basis of the current literature review. An additional search was conducted by the primary author (TTNL) to confirm studies identified by Honarvar et al. (2023) and identify new publications between January 6, 2023 and December 4, 2023. In addition to the search criteria described, candidate MRI predictors were confined to studies utilizing structural MRI; regional measurements identified in other modalities (e.g., functional MRI or functional connectivity) were not included. The search was further narrowed only to significant findings from longitudinal studies in which pre-drinking brain structures were independent variables forecasting subsequent adolescent alcohol use. Not considered were regional measurements related to alcohol use subsequent to drinking onset or cross-sectionally associated with adolescent alcohol use. To maintain fidelity with the original findings of prior work, the current study examined the same regions, laterality, and type of brain structure metric (volume, surface area, or thickness) that have previously been found to be statistically significant, which resulted in 31 regions of interest (ROI) measurements (see supplement). In total, 113 youth psychodevelopmental characteristic, cognition, brain structure, family, peers, and neighborhood domains from the NCANDA study were selected as candidate predictors following literature review (Fig. 3).

### 2.4 Data analyses

An overview of the analysis is provided in Fig. 3.

#### 2.4.1 Predictor reduction

Among the 113 candidate initial study-entry predictors, those with bivariate correlations of Pearson’s *r* ≥.80 with other variables suggested multicollinearity (Abu-Bader, 2010) and were eliminated. The correlation matrix was iteratively examined after removal of each predictor until no predictors showed correlations exceeding the *r* =.80 threshold. Brain regions were similarly examined for correlation among each other, using the same iterative procedure and threshold. Thirteen predictors were removed, resulting in a total of 100 candidate predictors (see supplemental spreadsheet). Prior to statistical analyses, all continuous (i.e., non-categorical) predictors were scaled (*M*=0.0; *SD*=1.0) to facilitate comparison among them.

#### 2.4.2 Part 1: Best subset of baseline precursors to alcohol use onset

Separately for each domain and outcome (i.e., First Drinking Onset or Regular Drinking Onset), the R package *glmulti* (R v.4.3.2 Calcagno and de Mazancourt, 2010; R Core Team, 2023) was used for automatic identification of the subset among the 100 candidate predictors that resulted in the optimal Cox Proportional Hazards (PH) model fit. Each Cox PH model was stratified according to the three recruitment age bands in line with NCANDA’s cohort sequential design (Brown et al., 2015) and covaried for age at study entry and sex. The inclusion of study site as a potential covariate did not change model results, directionality, or statistical significance and was omitted to maintain parsimony. The optimal model was defined according to the corrected Akaike Information Criterion (AICc; default setting of *glmulti*) and the search was performed by a genetic algorithm (*glmulti* argument method=g), as an exhaustive search among 2^100^ possible models (or predictor subsets) is computationally intractable. Fig. 3 lists the number of predictors for each domain and outcome that resulted from this search. Among those predictors that were identified in the best fitting model, only statistically significant predictors following multiple comparisons correction with a false discovery rate (FDR) of *q*=0.05 were interpreted. The input for FDR correction were *p-*values of all predictors in each best subset selection model, excluding covariates, pooled across results of six best subset selection domains for both First and Regular Drinking Onset (see supplement for full FDR correction results and the predictors included).

Note, we choose the Cox PH regression for this analysis as it models time to First and Regular Drinking Onset and uniquely handles censored samples (for explanation on survival models and censoring, see Clark et al., 2003). Herein, censored samples are youth that had not transitioned into first or regular drinking and, as such, their time to these events are unknown. Excluding these participants from analysis or assigning the time-to-event as the duration of data collection would bias results (Schober and Vetter, 2018); censoring allows for consideration of the full sample while accounting for transition status. In the present study, among N = 538 youth, 84 did not transition into drinking (i.e., alcohol naïve) and 167 drinkers did not transition into weekly drinking (i.e., remained infrequent drinkers).

Each optimal Cox PH model was assessed with bootstrap-based optimism correction (Harrell et al., 1996) using the R-package *rms* with 1000 bootstrap runs (Harrell Jr., 2023). The fit was assessed using the Craigg-Uhler/Nagelkerke pseudo R^2^ index (Nagelkerke, 1991) and Harrell’s Concordance Index (C-index; Harrell et al., 1982). Both metrics range from 0 to 1, with higher values indicating better model fit. The pseudo R^2^ is consistent with the classical ordinary least squares R^2^ and is interpreted as such in the present study. The C-Index is a goodness-of-fit metric that assesses censored survival models’ predictive ability. A C-Index of 0.5 indicates chance-level predictions, values closer to 1.0 suggest greater predictive ability, and values near 0.0 suggest below-chance predictive ability.

#### 2.4.3 Part 2: Prospective consequences of early alcohol use onset

Four robust regressions (*robustbase* R package; Maechler et al., 2023) examined the longitudinal relationship between: (1) First Drinking Onset and subsequent age of first binge drinking onset, (2) First Drinking Onset and subsequent age of weekly binge drinking onset, (3) Regular Drinking Onset and subsequent age of first binge drinking onset, and (4) Regular Drinking Onset and subsequent age of weekly binge drinking onset. The primary predictors of interest were First Drinking Onset (for models 1 and 2) and Regular Drinking Onset (models 3 and 4). All models controlled for age at study entry, sex, family history density of alcohol problems, parent education level, race, and ethnicity.

Two zero-inflated Poisson (ZIP) regressions (*pscl* R package; Zeileis et al., 2008) examined the longitudinal relationship between (1) First Drinking Onset and subsequent lifetime withdrawal symptoms and (2) Regular Drinking Onset and subsequent lifetime withdrawal symptoms. ZIP regressions were chosen to more accurately model count data with excessive zeroes. The primary predictors of interest were First Drinking Onset (for model 1) and Regular Drinking Onset (for model 2). All models included age at study entry, sex, family history density of alcohol problems, parent education level, race, and ethnicity as covariates. Age at study entry was included as a covariate to account for potential confounding effects of drinking duration on the number of lifetime withdrawal symptoms (e.g., youth who entered the study at younger ages would have more drinking years and thus greater opportunities for experiencing withdrawal effects). All model results were then corrected for multiple comparisons with Bonferroni correction, calculated as 0.05 divided by the number of predictors of interest tested across the six models (i.e., 0.05/6).

## 3 Results

### 3.1 Part 1: Best subset of baseline precursors to alcohol use onset

**Fig. 4:**
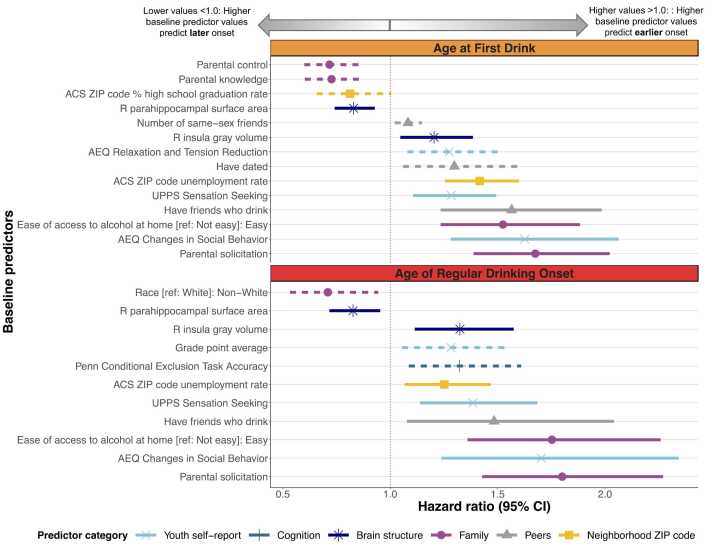
Results of best subsets selection using Cox Proportional Hazards models (Part 1: Best subset of baseline precursors to alcohol use onset). Hazard ratios and 95 % confidence intervals of Cox Proportional Hazards models forecasting two outcomes: 1) age at first drink (orange; top panel) and 2) age of regular drinking onset (red; bottom panel). Hazard ratios less than one indicate that higher baseline predictor values are associated with later drinking onset, whereas hazard values greater than one indicate that higher baseline predictor values are associated with earlier drinking onset. For ease of comparison, all continuous predictors have been scaled with mean of zero and standard deviation of one.

FDR corrected *p*-values and hazard ratios (HR) are reported below (see Fig. 4). Full model results, nominal *p*-values, and corrected *p*-values are provided in the supplement.

For categorical predictors, the reference group is shown in brackets. For categorical predictors with hazard ratios less than one, the group indicated, compared to the reference group, are more likely to initiate first or regular drinking later; the opposite interpretation applies for hazard ratios greater than one.

Solid lines denote baseline predictors that are statistically significantly associated with both age at first drink and age of regular drinking onset. Dashed lines indicate that predictors are uniquely associated with only one of the of two outcomes.

ACS: U.S. Census Bureau American Community Survey; AEQ: Alcohol Expectancies Questionnaire; L: Left; R: Right; TC: Total correct; UPPS: Urgency, Premeditation, Perseverance, Sensation Seeking Scale

#### 3.1.1 Age at first drink

##### 3.1.1.1 Youth psychodevelopmental characteristics

Of 28 baseline (i.e., at study entry) youth psychodevelopmental characteristic predictors, 10 emerged in the best fitting model (pseudo R^2^=.12; C-Index=.71). Greater baseline expectancies related to social behavioral changes following alcohol consumption (Changes in Social Behavior subscale, HR=1.63, adjusted *p*=.0001) and increased alcohol-induced relaxation expectancies (Relaxation and Tension subscale, HR=1.28, adjusted *p*=.02) predicted earlier First Drinking Onset (i.e., younger age of first drinking onset). Greater baseline sensation seeking tendencies (Sensation Seeking subscale, HR=1.29, adjusted *p*=.01) predicted earlier First Drinking Onset.

##### 3.1.1.2 Cognition

Of 12 baseline cognition predictors, five emerged in the best fitting model (pseudo R^2^=.02; C-Index=.66), none of which significantly predicted First Drinking Onset following multiple comparisons correction.

##### 3.1.1.3 Brain structure

Of 31 baseline brain structure predictors, five emerged in the best fitting model (pseudo R^2^=.04; C-Index=.68). Larger baseline right insula gray matter volume (HR=1.20, adjusted *p*=.03) and smaller right parahippocampal surface area (HR=.83, adjusted *p*=.01) predicted earlier First Drinking Onset.

##### 3.1.1.4 Family

Of 15 baseline family predictors, seven emerged in the best fitting model (pseudo R^2^=.11; C-Index=.69). Compared to youth who reported difficulty obtaining alcohol in the home, youth who reported easy access at baseline began drinking earlier (HR=1.52, adjusted *p*=.0001). Less baseline parental control (HR=.72, adjusted *p*=.003) and parental knowledge (HR=.72, adjusted *p*=.01) and more parental solicitation (HR=1.68, adjusted *p* =.0001) of youth activities, friendships, and whereabouts at baseline predicted earlier First Drinking Onset. Note that parental solicitation refers to the extent to which parents attempt to monitor youth (e.g., *How much do your parents TRY to know who your friends are?*; see supplement for full scale description) yet does not infer their knowledge or decision-making about youth activities.

##### 3.1.1.5 Peers

Of six baseline peer predictors, three emerged in the best fitting model (pseudo R^2^=.05; C-Index=.68). Compared to youth who never dated, those who dated by baseline began drinking earlier (HR=1.30, adjusted *p=*.04). Having more same-sex friends (HR=1.08, adjusted *p=*.03) and friends who drank (HR=1.57, adjusted *p=*.002) at baseline predicted earlier First Drinking Onset.

##### 3.1.1.6 Neighborhood

Of eight baseline neighborhood predictors, three emerged in the best fitting model (pseudo R^2^=.08; C-Index=.70). Residing in postal ZIP codes with fewer high school graduates (HR=.81, adjusted *p*= 01) and higher unemployment rates (HR=1.42, adjusted *p*=.0001) predicted earlier First Drinking Onset.

#### 3.1.2 Age of regular drinking onset

##### 3.1.2.1 Youth psychodevelopmental characteristics

Of 28 baseline youth psychodevelopmental characteristic predictors, eight emerged in the best fitting model (pseudo R^2^=.05; C-Index=.63). Greater baseline alcohol expectancies related to changes in social behavior (Changes in Social Behavior subscale, HR=1.70, adjusted *p=*.01), greater sensation seeking tendencies (Sensation Seeking subscale, HR=1.38, adjusted *p*=.01), and higher grade-point averages (HR=1.28, adjusted *p=*.04) predicted earlier Regular Drinking Onset.

##### 3.1.2.2 Cognition

Of 12 baseline cognition predictors, five emerged in the best fitting model (pseudo R^2^=.03; C-Index=.62). More correct responses on the Penn Conditional Exclusion Task (HR=1.32, adjusted *p=*.02) at baseline predicted earlier Regular Drinking Onset.

##### 3.1.2.3 Brain structure

Of 31 baseline brain structure predictors, six emerged in the best fitting model (pseudo R^2^=.04; C-Index=.66). Larger right insula gray volume (HR=1.32, adjusted *p=*.01) and smaller right parahippocampal surface area (HR=.83, adjusted *p=*.03) at baseline predicted earlier Regular Drinking Onset.

##### 3.1.2.4 Family

Of 15 baseline family predictors, six emerged in the best fitting model (pseudo R^2^=.10; C-Index=.67). Compared to youth who reported difficulty obtaining alcohol in the home, youth who reported easy access began regular drinking earlier (HR=1.75, adjusted *p=*.0001). More parental solicitation (HR=1.80, adjusted *p=*.0001) and being White, compared to youth who identified as non-White, predicted earlier Regular Drinking Onset.

##### 3.1.2.5 Peers

Of six peer baseline predictors, four emerged in the best fitting model (pseudo R^2^=.03; C-Index=.62). Having friends who drank (HR=1.48, adjusted *p=*.04) at baseline predicted earlier Regular Drinking Onset.

##### 3.1.2.6 Neighborhood

Of eight neighborhood baseline predictors, three emerged in the best fitting model (pseudo R^2^=.04; C-Index=.64). Residing in postal ZIP codes with higher unemployment rates (HR=1.25, adjusted *p* =.02) predicted earlier Regular Drinking Onset.

### 3.2 Part 2: Prospective consequences of early alcohol use onset

#### 3.2.1 Binge drinking onset

**Fig. 5:**
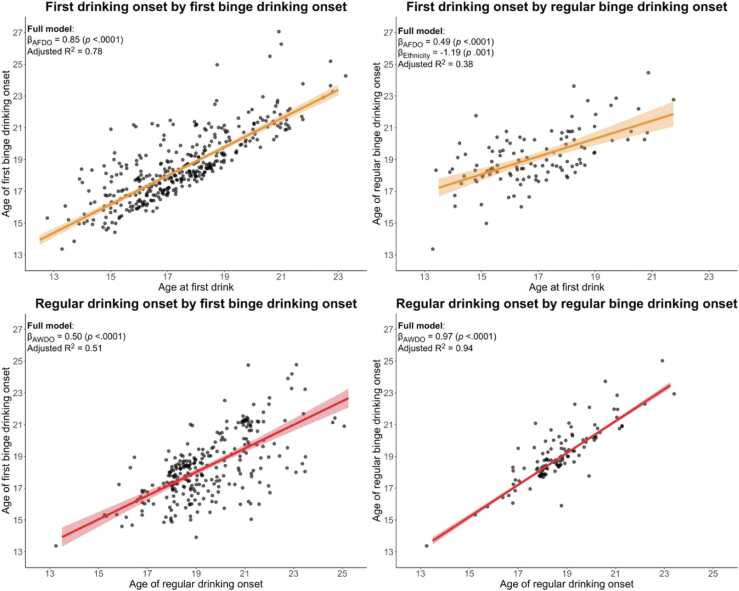
Scatter plot of relationship between adolescent drinking onset and binge drinking onset (Part 2: Prospective consequences of early alcohol use onset). Scatter plots show the bivariate relationship among two predictors of interest (age of first drinking onset age of regular drinking onset) and two outcomes of interest (age of binge drinking onset and age of weekly binge drinking onset). Four robust regressions examined the association between pairs of predictor and outcome, controlling for sex, family history density of alcohol problems, race, ethnicity, and parent educational achievement. The regression coefficient (β) for the predictor of interest and associated nominal *p*-values are indicated for each model. Only predictors statistically significant following multiple comparisons correction are shown. The amount of variance explained by the full model (R^2^) is also shown. Regression lines and 95 % confidence interval based on the slope and intercept of robust linear regression examining only the relationship between each predictor-outcome variable pair are shown in blue. To better visualize overlapping data points, a small amount of random variation in each point's x- and y-location was added in the graphs shown.

Controlling for covariates, younger First Drinking Onset was prospectively associated with both onset of earlier binge drinking (β=0.85, *p*<.0001) and onset of regular binge drinking (β=0.49, *p*<.0001) (Fig. 5). Non-Hispanic youth were more likely to engage in regular binge drinking earlier (β= −1.19, *p*=.001). Other sociodemographic factors did not significantly predict outcomes following multiple comparisons correction.

Controlling for covariates, younger Regular Drinking Onset was prospectively associated with earlier binge drinking (β=0.50, *p*<.0001) and regular binge drinking (β=.97, *p*<.0001) onset (Fig. 5). Sociodemographic factors were not statistically significant following multiple comparisons correction.

#### 3.2.2 Lifetime withdrawal symptoms

Incidence rate ratios (IRRs) and *p*-values are reported below. IRRs were calculated by exponentiating regression coefficients and interpreted similarly to odds ratios. Full results of ZIP regressions forecasting subsequent withdrawal symptoms as a function of ages of first and regular drinking onset are reported in the supplement.

**Fig. 6:**
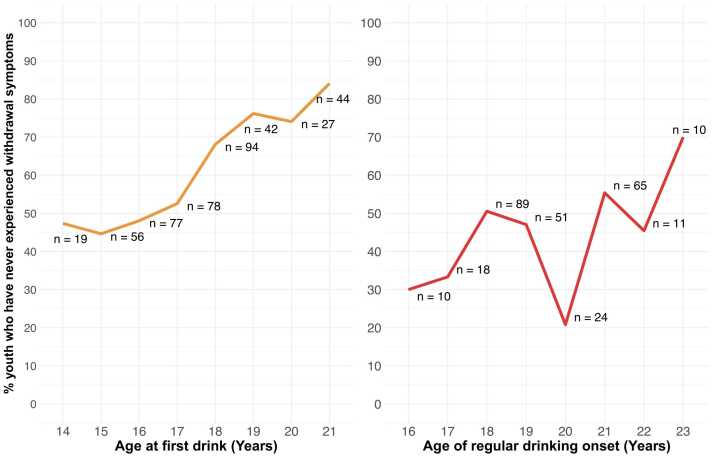
Percent of youth who have never experienced withdrawal symptoms by onset ages (Part 2: Prospective consequences of early alcohol use onset). The percent of youth who have never experienced withdrawal symptoms by at each age of first drink (left) and regular drinking onset (right) are shown. The total number of participants (n) at each age are shown next to each data point; data points in which n <10 are suppressed and not shown in the figure.

Controlling for all covariates, in the Poisson count model (i.e., among youth who have experienced at least one withdrawal symptom), First Drinking Onset was not associated with the number of lifetime withdrawal symptoms (*p*>.05). In the logit model (i.e., among youth who have never experienced any withdrawal symptoms), a one-year delay in First Drinking Onset increased the odds of never experiencing any withdrawal symptoms by 62 % (IRR= 1.62, *p*<.0001; Fig. 6).

Controlling for all covariates, Regular Drinking Onset was not associated with the number of lifetime withdrawal symptoms (*p*>.05) in the Poisson count model. In the logit model, a delay of one year in Regular Drinking Onset increased the odds of never experiencing any withdrawal symptoms by 29% (IRR=1.29, *p* =.004; Fig. 6). For both First and Regular Drinking Onset, covariates (sex, family history density of alcohol problems, parent educational attainment, race, ethnicity, and age at study onset) were not statistically significant following multiple comparisons correction.

## 4 Discussion

This study used longitudinal, prospective data to identify baseline precursors (i.e., at study entry) and outcomes of adolescent alcohol use onset in a two-part analysis. Part 1 leveraged automated variable selection (i.e., best subset selection) to identify the most robust initial predictors of first drinking onset and regular drinking onset among 100 pre-drinking characteristics. Part 2 assessed the clinical utility of first and regular drinking onset in forecasting subsequent binge drinking onset and post-drinking (e.g., hangover and withdrawal) symptoms.

### 4.1 Part 1: Best subset of baseline precursors to alcohol use onset

In Part 1, it was hypothesized that variables identified in previous studies that were predictive of adolescent alcohol *use* patterns would also emerge as the most robust prospective predictors of adolescent drinking *onset*. Results showed that participants who were most likely to transition into first and regular drinking earlier had, at baseline: higher sensation seeking dispositions, higher expectations of changes in social behavior as a result of drinking, higher parental solicitation, lower parental control, lower parental knowledge, greater access to alcohol at home, and more friends who drink. Sociodemographic characteristics of the ZIP code youth resided in appeared to play a role in forecasting both first and regular drinking onset, such that youth who resided in ZIP codes with higher unemployment rates and lower high school graduation rates were more likely to transition earlier. Overall, current findings are generally consistent with previous studies that reported associations between age of first drinking onset with peer drinking patterns (Fisher et al., 2007), alcohol expectancies (Bekman et al., 2010, Fisher et al., 2007), and substance availability (Trujillo et al., 2019).

Interestingly, more baseline parental solicitation was one of the strongest precursors to initiation and predicted *earlier* onset, whereas more parental control and knowledge predicted *later* onset. As noted, parental solicitation refers to the extent to which parents attempt to monitor youth and does not infer their knowledge or decision-making about youth activities. Parental solicitation, control, and knowledge represent aspects of parental monitoring. Other metrics of parental monitoring assessed in this study – parental warmth and supervision – were not found to be robust initial predictors of alcohol use onset in this sample. While parental monitoring has been operationalized in various ways (Hardie, 2021), it was most recently conceptualized as an action- and goal-oriented “set of correlated parenting behaviors involving attention to and tracking of the child’s whereabouts, activities, and adaptations.” (Dishion and McMahon, 1998) Parental monitoring, and the individual actions therein (e.g., solicitation), are adaptive through a feedback loop of monitoring, evaluation, and behavioral adjustments guided by parents’ goals for the youth’s development. Throughout adolescence, goals may include increased behavioral control, passive parenting, or guided nurturing (Hardie, 2021). As age was statistically controlled for in the present study’s analyses, additional research is needed to understand the dynamic changes in parental monitoring behaviors across developmental age ranges and their interactions with adolescent alcohol use onset. Nevertheless, results suggest that, regardless of age, actual knowledge and involvement in youth behaviors appear to be key protective factors against early drinking onset. It may be that parental solicitation increases when youth are at greater risk of drinking (e.g., have peers who drink); or perhaps parental solicitation serves as a proxy for parental concern, and higher levels of solicitation suggests greater parent concern about youth behaviors. Another possibility is that parental solicitation may reflect parent-child relationship strength. However, further research is needed to elucidate these and other underlying mechanisms between parenting and alcohol use onset. Overall, findings on parental involvement are in line with other studies that found increased parental control and parental knowledge, sometimes also referred to as parental monitoring, serve as protective factors against alcohol and other substance use (Bray et al., 2022, Mills et al., 2021, Sellers et al., 2018). Parent solicitation, on the other hand, has been associated with *more* alcohol use (Alexander et al., 2023, Fletcher et al., 2004) as seen here.

Selection of brain structures measured at baseline for predicting drinking onset variables was based solely on published findings from longitudinal studies that reported specific regions as predictive of drinking in adolescents. Of the 31 regions used in the analysis herein, only two measures, smaller right insula volume and larger parahippocampal gyrus area, predicted first and regular drinking onset. These findings are in line with the known literature, as both neurostructural findings are regions frequently associated with alcohol use (Koob and Volkow, 2010). The insula is a multifunctional structure situated within the lateral sulcus that plays critical roles in perception, subjective emotional processing, social cognition, risk-reward decision making, and attention (Uddin et al., 2017). The parahippocampal gyrus and associated hippocampal region are implicated in reward processing and learning and memory. Insular activation has been found to increase in response to alcohol cues and during alcohol consumption (Campbell and Lawrence, 2021, Manuweera et al., 2022) and decreased hippocampal and parahippocampal volumes have been associated with risky drinking behaviors (Heikkinen et al., 2017, Hua et al., 2020, Meda et al., 2018). Use of pre-identified brain metrics narrowed the number of variables for testing in prediction models and also tested replication (Hyatt et al., 2020). In large part, the brain regions examined from the literature were not replicated as drinking precursors; the two that emerged as significant drinking predictors were unilateral and in opposing directions, raising the possibility that they were chance occurrences. Notably, these brain measures were weaker predictors than several behavioral and environmental variables, such as psychodevelopmental characteristics, peer, family, and neighborhood characteristics. Surprisingly, neither did frontal regions survive multiple comparisons correction nor did three other factors previously shown to be correlated with adolescent drinking emerge among the strongest predictors of drinking onset: sleep patterns or chronotype (Hasler et al., 2022, Hasler et al., 2015), internalizing and externalizing traits (Hardee et al., 2018), and geolocated alcohol outlet density (Chen et al., 2010).

### 4.2 Part 2: Prospective consequences of early alcohol use onset

A strong body of research shows a correspondence between earlier alcohol use initiation and potential deleterious consequences. The present study examined both first and regular drinking onset to disentangle if, and how, each differs in its predictive utility in forecasting subsequent binge drinking initiation and withdrawal symptoms. Unexpectedly, both metrics showed comparable predictive strengths with binge drinking onset. Indeed, the age at which youth initiate drinking exhibited a *stronger* association with risk of experiencing withdrawal symptoms than the age at which youth initiate regular, or habitual drinking. Among youth who had never experienced any withdrawal symptoms, a one-year delay in first drinking initiation predicted a 62 % increased odds of never experiencing any withdrawal symptoms compared with a 29 % increased odds of never experiencing any withdrawal symptoms with each year delay in regular drinking initiation. A potential reason for this observation is that the prospective design more accurately captured ages of onset, eliminating reliance on retrospective recall. Prior longitudinal studies have found a tendency to report older ages of initiation with more time from initiation (Golub et al., 2000). It is possible that the attenuated impact of first drinking onset found in prior studies reflects greater recall bias for the exact age of first drink, as youth may be more likely to recall their first intoxication rather than their year of first drink. Additionally, the present study defined first drinking onset as age of first full standard drink, and results may differ for age of first sip. Overall, results highlight the importance of delaying alcohol use initiation, not just regular drinking, among adolescents as a potential mechanism to mitigate potential downstream deleterious alcohol-related effects (Guttmannova et al., 2012).

### 4.3 Limitations

Despite the many strengths of this NCANDA-based study, including its prospective longitudinal design and large nationwide socio-demographically diverse sample, several limitations are of note. Firstly, while the number and types of predictors examined in Part 1 are comprehensive, they are not exhaustive. For example, future studies should investigate brain-wide regional metrics as predictors of drinking trajectories. Further, data on several key correlates of adolescent alcohol use were not available on all participants and thus were not modeled, including childhood trauma (Sartor et al., 2013), sexual orientation (Marshal et al., 2008), gender identity (Kann et al., 2016), and family attitudes towards minority sexual identities (Fish et al., 2020).

Part 1 analyses followed a theory and data-informed approach, and Part 2 focused on examining the relationship between alcohol use onset, binge drinking onset, and withdrawal symptoms. While the analytic approaches in Part 2 adequately addressed the research question and elucidated the relationships among the three key metrics of interest, other potential contributing factors to binge drinking onset and withdrawal effects were not modeled. For example, it is plausible that the predictor domains examined in Part 1 also contribute to heavy episodic drinking and withdrawal experiences among youth, above and beyond age of drinking onset. This possibility should be considered when interpreting present results and may be explored in future research examining prospective predictors of binge drinking, withdrawal symptoms, and other alcohol-related consequences.

Statistically, a key limitation was that the present study used bootstrapping to assess model bias and overfitting rather than attempting to replicate the results in an independent sample. This consideration is especially important given the large number of variables examined. Additionally, a limitation of best subsets selection is that the resulting best model is dependent on the candidate predictors entered into the regression, as the model is optimized based on the combination of predictors that maximizes goodness-of-fit indices. Although evidence suggests best subset selection performs comparably to other commonly used variable selection techniques (Hastie et al., 2020, Hastie et al., 2017), the best fitting model is selected in a data-driven atheoretical process that may be susceptible to multicollinearity effects. To account for these known weaknesses, the present study used a theory-driven approach for selecting candidate predictors and omitted highly correlated predictors prior to analysis.

In considering the generalizability of results, sample selection and exclusionary criteria are important. Of 831 participants in the NCANDA study, 32 % initiated alcohol use prior to study entry and were thus excluded due to lack of pre-drinking predictor information. Compared to the overall NCANDA sample, participants included in analyses were younger, more likely from the 12 – 14 and 15 – 17 recruitment bands, and transitioned into alcohol use at a later age. This difference, while meaningful, is not surprising, as youth who were recruited at younger ages were more likely to transition while enrolled in the study. The mean age of transition in the present study was 17.8 years old, compared to the national mean of 17.1 years old (National Survey on Drug Use and Health, 2022). Thus, caution is needed in generalizing findings to the larger NCANDA cohort and to youth with younger or older onset ages, and the exclusion of youth who initiated drinking prior to study entry may also limit generalizability to youth nationally based on age of onset. Further, given the changes in adolescence, interpretations of predictors found to be associated with age of first and regular drinking onset are limited to the age ranges examined in the present study, and may differ for younger and older ages of onset.

### 4.4 Translational value of identifying predictors of youthful drinking

The current study offers potential clinically feasible intervention targets to delay first and regular drinking onset, which in turn may lower risks of problematic drinking behavior and subsequent deleterious alcohol-related outcomes later in life. At the youth level, increased alcohol expectancies related to changes in social behavior were among the most robust predictors of earlier first and weekly drinking onset. A potential intervention would entail modifying youth expectancies, a target of the Alcohol Literacy Challenge, a single-session group intervention for adolescents and young adults (Fried and Dunn, 2012). At the family level, salient precursors related to parental involvement (parental knowledge, control, and solicitation) may be targeted using evidence-based interventions such as the Parent Management Training – Oregon Model (Forgatch and Kjøbli, 2016). Increased parent involvement not only intervenes at the family-level, but may have implications for peer relationships, as greater parent involvement has been found to correlate with lower peer alcohol use norms (Handren et al., 2016). Finally, the predictive relationship between neighborhood economic security and number of alcohol-related establishments (i.e., liquor stores and bars/pubs) and their proximity to homes and schools and youth drinking onset may be valuable considerations for policymakers in future substance use-related initiatives.

## 5 Conclusion

The results of this prospective, longitudinal study have notable translational value. First, pre-drinking characteristics related to youth psychodevelopmental characteristics, cognition, brain structure, family, peers, and neighborhood were all significant predictors of first and regular drinking onset. Among these domains, youth personality and disposition and parental behaviors related to solicitation, control, and knowledge of youth activities and whereabouts appear to be most influential. Pre-drinking neuroanatomy, albeit highly restrictive, had low predictive value of the drinking styles examined herein. Second, precursors to first and regular drinking onset may overlap, but are not identical, underscoring their dissociable nature. Third, although both first and regular drinking onset were each strongly predictive of subsequent risky drinking behaviors, first drinking onset may be a more useful metric of future use than previously thought (Kuntsche et al., 2016). Critically, the prospective design of these NCANDA data enabled accurate determination of drinking onset ages and transition time to initiation and regular drinking. Consequently, the current study lays a foundational model for future explorations of adolescent drinking initiation with other large-scale national studies such as the Adolescent Brain Cognition Development (ABCD) study to examine additional predictors and complex interactions among them.

## Data statement

Data presented in this study were drawn from the ongoing National Consortium on Alcohol and NeuroDevelopment in Adolescence (NCANDA) study. The released dataset with all participants is publicly available on the National Institute of Mental Health Data Archive Collection C4513. The authors do not have permission to share data otherwise.

## Funding

This work was supported by the U.S. National Institute on Drug Abuse (R01 DA057567 [MPIs: Pohl & Tapert]) and the Institute on Alcohol Abuse and Alcoholism (U01 AA021681 [PI: Goldston], U01 AA021690 [MPIs: Clark & Luna], U01 AA021691 [PI: Nagel], U01 AA021692 [PI: Tapert], U24 AA021695 [MPIs: Tapert & Brown], U01 AA021696 [MPIs: Baker & Muller-Oehring], U24 AA021697 [MPIs: Pohl & Pfefferbaum], and the Patricia A. Judd and Family Endowed Post-Doctoral Fellowship in ADHD Research.

## CRediT authorship contribution statement

**Edith V. Sullivan:** Writing – review & editing, Writing – original draft, Validation, Supervision, Project administration, Funding acquisition, Conceptualization. **Wesley K. Thompson:** Writing – review & editing, Writing – original draft, Software, Methodology. **Massimiliano de Zambotti:** Writing – review & editing, Project administration, Funding acquisition. **Adolf Pfefferbaum:** Writing – review & editing, Writing – original draft, Validation, Supervision, Project administration, Funding acquisition, Conceptualization. **Brian Knutson:** Writing – review & editing. **David B. Goldston:** Writing – review & editing, Project administration, Funding acquisition. **Fiona C. Baker:** Writing – review & editing, Project administration, Funding acquisition. **Bonnie J. Nagel:** Writing – review & editing, Project administration, Funding acquisition. **Tam T. Nguyen-Louie:** Writing – review & editing, Writing – original draft, Visualization, Validation, Software, Methodology, Investigation, Formal analysis, Data curation, Conceptualization. **Kate B. Nooner:** Writing – review & editing, Project administration, Funding acquisition. **Beatriz Luna:** Writing – review & editing, Project administration, Funding acquisition. **Sonja C. Eberson-Shumate:** Writing – review & editing, Validation, Resources, Project administration, Data curation. **Susan F. Tapert:** Writing – review & editing, Writing – original draft, Visualization, Supervision, Resources, Project administration, Methodology, Funding acquisition, Data curation, Conceptualization. **Camila Gonzalez:** Writing – review & editing, Validation. **Kilian M. Pohl:** Writing – review & editing, Writing – original draft, Visualization, Validation, Supervision, Resources, Project administration, Methodology, Funding acquisition, Data curation, Conceptualization. **Duncan B. Clark:** Writing – review & editing, Project administration, Funding acquisition. **Natasha E. Wade:** Writing – review & editing, Validation.

## Declaration of Competing Interest

The authors declare that they have no known competing financial interests or personal relationships that could have appeared to influence the work reported in this paper. Unrelated to the work reported in this paper, Massimiliano de Zambotti reports a relationship with Noctrix Health Inc. that includes: funding grants. Massimiliano de Zambotti reports a relationship with Verily Life Sciences LLC that includes: funding grants. Massimiliano de Zambotti reports a relationship with Honda Motor Co Ltd that includes: funding grants. Massimiliano de Zambotti reports a relationship with Lisa Health, Inc that includes: employment and equity or stocks. If there are other authors, they declare that they have no known competing financial interests or personal relationships that could have appeared to influence the work reported in this paper.

## Data Availability

The authors do not have permission to share data.
