# Supplementary material for: Multi-dimensional predictors of first drinking initiation and regular drinking onset in adolescence: A prospective longitudinal study
Source: Dev Cogn Neurosci. 2024 Jul 30;69:101424. doi: 10.1016/j.dcn.2024.101424 (PMC11342118; doi:10.1016/j.dcn.2024.101424)
Supplement: Supplementary file 1 — Supplementary material [file mmc1.docx]

**Declaration of Competing Interest**

The authors declare that they have no known competing financial interests or personal relationships that could have appeared to influence the work reported in this paper. MdZ has received research funding unrelated to this work from Noctrix Health Inc., Verily Life Science LLC, and Honda Motor Co., Ltd. MdZ is a co-founder and Chief Scientific Officer at Lisa Health Inc. and has ownership of shares in Lisa Health Inc.

**References**

Abu-Bader, S.H., 2010. Advanced and multivariate statistical methods for social science research. Oxford University Press.

Achenbach, T.M., 1991. Manual for the youth self-report and 1991 profile. University of Vermont Department of Psychiatry.

Achenbach, T.M., Rescorla, L.A., 2003. Manual for the ASEBA adult forms & profiles. Burlington, VT: University of Vermont, Research Center for Children, Youth and Families, Burlington, VT.

Alexander, J.D., Freis, S.M., Zellers, S.M., Corley, R., Ledbetter, A., Schneider, R.K., Phelan, C., Subramonyam, H., Frieser, M., Rea-Sandin, G., Stocker, M.E., Vernier, H., Jiang, M., Luo, Y., Zhao, Q., Rhea, S.A., Hewitt, J., Luciana, M., McGue, M., Wilson, S., Resnick, P., Friedman, N.P., Vrieze, S.I., 2023. Evaluating longitudinal relationships between parental monitoring and substance use in a multi-year, intensive longitudinal study of 670 adolescent twins. Front. Psychiatry 14. https://doi.org/10.3389/fpsyt.2023.1149079

Armenta, B.E., Sittner, K.J., Whitbeck, L.B., 2016. Predicting the Onset of Alcohol Use and the Development of Alcohol Use Disorder Among Indigenous Adolescents. Child Dev. 87, 870–882. https://doi.org/10.1111/cdev.12506

Bachman, J.G., 1981. Monitoring the Future: Questionnaire Responses from the Nation’s High School Seniors, 1980. ERIC.

Baranger, D.A.A., Demers, C.H., Elsayed, N.M., Knodt, A.R., Radtke, S.R., Desmarais, A., Few, L.R., Agrawal, A., Heath, A.C., Barch, D.M., Squeglia, L.M., Williamson, D.E., Hariri, A.R., Bogdan, R., 2020. Convergent evidence for predispositional effects of brain gray matter volume on alcohol consumption. Biol. Psychiatry 87, 645–655. https://doi.org/10.1016/j.biopsych.2019.08.029

Bekman, N.M., Cummins, K., Brown, S.A., 2010. Affective and Personality Risk and Cognitive Mediators of Initial Adolescent Alcohol Use. J. Stud. Alcohol Drugs 71, 570–580. https://doi.org/10.15288/jsad.2010.71.570

Bertsimas, D., King, A., Mazumder, R., 2016. Best subset selection via a modern optimization lens. Ann. Stat. 44, 813–852. https://doi.org/10.1214/15-AOS1388

Bray, J.H., Gallegos, M.I., Cain, M.K., Zaring-Hinkle, B., 2022. Parental monitoring, family conflict, and adolescent alcohol use: A longitudinal latent class analysis. J. Fam. Psychol. 36, 1154–1160. https://doi.org/10.1037/fam0001019

Bronfenbrenner, U., 1974. Developmental Research, Public Policy, and the Ecology of Childhood. Child Dev. 45, 1–5. https://doi.org/10.2307/1127743

Bronfenbrenner, U., Ceci, S.J., 1994. Nature-nuture reconceptualized in developmental perspective: A bioecological model. Psychol. Rev. 101, 568–586. https://doi.org/10.1037/0033-295X.101.4.568

Brown, S.A., Brumback, T., Tomlinson, K., Cummins, K., Thompson, W.K., Nagel, B.J., De Bellis, M.D., Hooper, S.R., Clark, D.B., Chung, T., Hasler, B.P., Colrain, I.M., Baker, F.C., Prouty, D., Pfefferbaum, A., Sullivan, E.V., Pohl, K.M., Rohlfing, T., Nichols, B.N., Chu, W., Tapert, S.F., 2015. The National Consortium on Alcohol and NeuroDevelopment in Adolescence (NCANDA): A Multisite Study of Adolescent Development and Substance Use. J. Stud. Alcohol Drugs 76, 895–908. https://doi.org/10.15288/jsad.2015.76.895

Brown, S.A., Christiansen, B.A., Goldman, M.S., 1987. The Alcohol Expectancy Questionnaire: an instrument for the assessment of adolescent and adult alcohol expectancies. J. Stud. Alcohol 48, 483–491. https://doi.org/10.15288/jsa.1987.48.483

Brown, S.A., Myers, M.G., Lippke, L., Tapert, S.F., Stewart, D.G., Vik, P.W., 1998. Psychometric evaluation of the Customary Drinking and Drug Use Record (CDDR): a measure of adolescent alcohol and drug involvement. J. Stud. Alcohol 59, 427–438. https://doi.org/10.15288/jsa.1998.59.427

Brumback, T.Y., Worley, M., Nguyen-Louie, T.T., Squeglia, L.M., Jacobus, J., Tapert, S.F., 2016. Neural predictors of alcohol use and psychopathology symptoms in adolescents. Dev. Psychopathol. 28, 1209–1216. https://doi.org/10.1017/S0954579416000766

Calcagno, V., de Mazancourt, C., 2010. glmulti: An R Package for Easy Automated Model Selection with (Generalized) Linear Models. Journal of Statistical Software 34, 1–29. https://doi.org/10.18637/jss.v034.i12

Campbell, E.J., Lawrence, A.J., 2021. It’s more than just interoception: The insular cortex involvement in alcohol use disorder. J. Neurochem. 157, 1644–1651. https://doi.org/10.1111/jnc.15310

Carskadon, M.A., Acebo, C., 1993. A self-administered rating scale for pubertal development. J. Adolesc. Health Off. Publ. Soc. Adolesc. Med. 14, 190–195. https://doi.org/10.1016/1054-139x(93)90004-9

Cheetham, A., Allen, N.B., Whittle, S., Simmons, J., Yücel, M., Lubman, D.I., 2014. Volumetric differences in the anterior cingulate cortex prospectively predict alcohol-related problems in adolescence. Psychopharmacology (Berl.) 231, 1731–1742. https://doi.org/10.1007/s00213-014-3483-8

Chen, M.-J., Grube, J.W., Gruenewald, P.J., 2010. Community alcohol outlet density and underage drinking. Addict. Abingdon Engl. 105, 270–278. https://doi.org/10.1111/j.1360-0443.2009.02772.x

Clark, T.G., Bradburn, M.J., Love, S.B., Altman, D.G., 2003. Survival analysis part I: Basic concepts and first analyses. Br. J. Cancer 89, 232–238. https://doi.org/10.1038/sj.bjc.6601118

Connor, J.P., Weier, M., Hall, W.D., 2019. The Age of Onset of Alcohol Use Disorders, in: de Girolamo, G., McGorry, P.D., Sartorius, N. (Eds.), Age of Onset of Mental Disorders: Etiopathogenetic and Treatment Implications. Springer International Publishing, Cham, pp. 169–182. https://doi.org/10.1007/978-3-319-72619-9_9

Cyders, M.A., Smith, G.T., Spillane, N.S., Fischer, S., Annus, A.M., Peterson, C., 2007. Integration of impulsivity and positive mood to predict risky behavior: Development and validation of a measure of positive urgency. Psychol. Assess. 19, 107–118. https://doi.org/10.1037/1040-3590.19.1.107

Dawson, D.A., Goldstein, R.B., Patricia Chou, S., June Ruan, W., Grant, B.F., 2008. Age at first drink and the first incidence of adult-onset DSM-IV alcohol use disorders. Alcohol. Clin. Exp. Res. 32, 2149–2160. https://doi.org/10.1111/j.1530-0277.2008.00806.x

DeWit, D.J., Adlaf, E.M., Offord, D.R., Ogborne, A.C., 2000. Age at first alcohol use: A risk factor for the development of alcohol disorders. Am. J. Psychiatry 157, 745–750. https://doi.org/10.1176/appi.ajp.157.5.745

Dishion, T.J., McMahon, R.J., 1998. Parental monitoring and the prevention of child and adolescent problem behavior: A conceptual and empirical formulation. Clin. Child Fam. Psychol. Rev. 1, 61–75.

Farmer, R.F., Gau, J.M., Seeley, J.R., Kosty, D.B., Sher, K.J., Lewinsohn, P.M., 2016. Internalizing and externalizing disorders as predictors of alcohol use disorder onset during three developmental periods. Drug Alcohol Depend. 164, 38–46. https://doi.org/10.1016/j.drugalcdep.2016.04.021

Fish, J.N., Russell, B.S., Watson, R.J., Russell, S.T., 2020. Parent-child relationships and sexual minority youth: Implications for adult alcohol abuse. J. Youth Adolesc. 49, 2034–2046. https://doi.org/10.1007/s10964-020-01299-7

Fisher, L.B., Miles, I.W., Austin, S.B., Camargo, C.A., Jr, Colditz, G.A., 2007. Predictors of initiation of alcohol use among us adolescents: Findings from a prospective cohort study. Arch. Pediatr. Adolesc. Med. 161, 959–966. https://doi.org/10.1001/archpedi.161.10.959

Fletcher, A.C., Steinberg, L., Williams-Wheeler, M., 2004. Parental influences on adolescent problem behavior: Revisiting Stattin and Kerr. Child Dev. 75, 781–796. https://doi.org/10.1111/j.1467-8624.2004.00706.x

Forgatch, M.S., Kjøbli, J., 2016. Parent management training-Oregon model: Adapting intervention with rigorous research. Fam. Process 55, 500–513. https://doi.org/10.1111/famp.12224

Fried, A.B., Dunn, M.E., 2012. The Expectancy Challenge Alcohol Literacy Curriculum (ECALC): A single session group intervention to reduce alcohol use. Psychol. Addict. Behav. J. Soc. Psychol. Addict. Behav. 26, 615–620. https://doi.org/10.1037/a0027585

Gioia, G.A., Isquith, P.K., Retzlaff, P.D., Espy, K.A., 2002. Confirmatory factor analysis of the Behavior Rating Inventory of Executive Function (BRIEF) in a clinical sample. Child Neuropsychol. J. Norm. Abnorm. Dev. Child. Adolesc. 8, 249–257. https://doi.org/10.1076/chin.8.4.249.13513

Golub, A., Johnson, B.D., Labouvie, E., 2000. On correcting biases in self-reports of age at first substance use with repeated cross-section analysis. J. Quant. Criminol. 16, 45–68. https://doi.org/10.1023/A:1007573411129

Gosling, S.D., Rentfrow, P.J., Swann, W.B., 2003. A very brief measure of the Big-Five personality domains. J. Res. Personal. 37, 504–528. https://doi.org/10.1016/S0092-6566(03)00046-1

Grant, B.F., Dawson, D.A., 1997. Age at onset of alcohol use and its association with DSM-IV alcohol abuse and dependence: results from the National Longitudinal Alcohol Epidemiologic Survey. J. Subst. Abuse 9, 103–110. https://doi.org/10.1016/s0899-3289(97)90009-2

Gruber, E., DiClemente, R.J., Anderson, M.M., Lodico, M., 1996. Early drinking onset and its association with alcohol use and problem behavior in late adolescence. Prev. Med. 25, 293–300. https://doi.org/10.1006/pmed.1996.0059

Gur, R.C., Richard, J., Hughett, P., Calkins, M.E., Macy, L., Bilker, W.B., Brensinger, C., Gur, R.E., 2010. A cognitive neuroscience-based computerized battery for efficient measurement of individual differences: Standardization and initial construct validation. J. Neurosci. Methods 187, 254–262. https://doi.org/10.1016/j.jneumeth.2009.11.017

Guttmannova, K., Hill, K.G., Bailey, J.A., Lee, J.O., Hartigan, L.A., Hawkins, J.D., Catalano, R.F., 2012. Examining explanatory mechanisms of the effects of early alcohol use on young adult alcohol dependence. J. Stud. Alcohol Drugs 73, 379–390. https://doi.org/10.15288/jsad.2012.73.379

Handren, L.M., Donaldson, C.D., Crano, W.D., 2016. Adolescent alcohol use: Protective and predictive parent, peer, and self-related factors. Prev. Sci. Off. J. Soc. Prev. Res. 17, 862–871. https://doi.org/10.1007/s11121-016-0695-7

Hardee, J.E., Cope, L.M., Martz, M.E., Heitzeg, M.M., 2018. Review of neurobiological influences on externalizing and internalizing pathways to alcohol use disorder. Curr. Behav. Neurosci. Rep. 5, 249–262. https://doi.org/10.1007/s40473-018-0166-5

Hardie, B., 2021. Reconceptualising parental monitoring within a model of goal-directed parental action. New Ideas Psychol. 61, 100847. https://doi.org/10.1016/j.newideapsych.2020.100847

Harrell, F.E., Jr, Califf, R.M., Pryor, D.B., Lee, K.L., Rosati, R.A., 1982. Evaluating the yield of medical tests. JAMA 247, 2543–2546. https://doi.org/10.1001/jama.1982.03320430047030

Harrell, F.E., Lee, K.L., Mark, D.B., 1996. Multivariable prognostic models: issues in developing models, evaluating assumptions and adequacy, and measuring and reducing errors. Stat. Med. 15, 361–387. https://doi.org/10.1002/(SICI)1097-0258(19960229)15:4<361::AID-SIM168>3.0.CO;2-4

Harrell Jr., F.E., 2023. _rms: Regression Modeling Strategies_. R package version 6.7-1.

Hasler, B.P., Graves, J.L., Wallace, M.L., Claudatos, S., Franzen, P.L., Nooner, K.B., Brown, S.A., Tapert, S.F., Baker, F.C., Clark, D.B., 2022. Self-reported sleep and circadian characteristics predict alcohol and cannabis use: A longitudinal analysis of the National Consortium on Alcohol and Neurodevelopment in Adolescence study. Alcohol. Clin. Exp. Res. 46, 848–860. https://doi.org/10.1111/acer.14808

Hasler, B.P., Soehner, A.M., Clark, D.B., 2015. Sleep and circadian contributions to adolescent alcohol use disorder. Alcohol, Special Issue: Sleep, Circadian Rhythms and Alcohol 49, 377–387. https://doi.org/10.1016/j.alcohol.2014.06.010

Hastie, T., Tibshirani, R., Tibshirani, R.J., 2017. Extended comparisons of best subset selection, forward stepwise selection, and the lasso. ArXiv Prepr. ArXiv170708692.

Hastie, T., Tibshirani, Robert, Tibshirani, Ryan, 2020. Best subset, forward stepwise or lasso? Analysis and recommendations based on extensive comparisons. Stat. Sci. 35, 579–592. https://doi.org/10.1214/19-STS733

Hatoum, A.S., Johnson, E.C., Baranger, D.A.A., Paul, S.E., Agrawal, A., Bogdan, R., 2021. Polygenic risk scores for alcohol involvement relate to brain structure in substance-naïve children: Results from the ABCD study. Genes Brain Behav. 20, e12756. https://doi.org/10.1111/gbb.12756

Heikkinen, N., Niskanen, E., Könönen, M., Tolmunen, T., Kekkonen, V., Kivimäki, P., Tanila, H., Laukkanen, E., Vanninen, R., 2017. Alcohol consumption during adolescence is associated with reduced grey matter volumes. Addict. Abingdon Engl. 112, 604–613. https://doi.org/10.1111/add.13697

Hill, S.Y., O’Brien, J., 2015. Psychological and neurobiological precursors of alcohol use disorders in high risk youth. Curr. Addict. Rep. 2, 104–113. https://doi.org/10.1007/s40429-015-0051-1

Hingson, R.W., Heeren, T., Winter, M.R., 2006. Age at drinking onset and alcohol dependence: age at onset, duration, and severity. Arch. Pediatr. Adolesc. Med. 160, 739–746. https://doi.org/10.1001/archpedi.160.7.739

Hingson, R.W., Zha, W., 2009. Age of drinking onset, alcohol use disorders, frequent heavy drinking, and unintentionally injuring oneself and others after drinking. Pediatrics 123, 1477–1484. https://doi.org/10.1542/peds.2008-2176

Honarvar, F., Arfaie, S., Edalati, H., Ghasroddashti, A., Solgi, A., Mashayekhi, M.S., Mofatteh, M., Ren, L.Y., Kwan, A.T.H., Keramatian, K., 2023. Neuroanatomical predictors of problematic alcohol consumption in adolescents: A systematic review of longitudinal studies. Alcohol Alcohol. Oxf. Oxfs. 58, 455–471. https://doi.org/10.1093/alcalc/agad049

Hua, J.P.Y., Piasecki, T.M., McDowell, Y.E., Boness, C.L., Trela, C.J., Merrill, A.M., Sher, K.J., Kerns, J.G., 2020. Alcohol use in young adults associated with cortical gyrification. Drug Alcohol Depend. 209, 107925. https://doi.org/10.1016/j.drugalcdep.2020.107925

Hyatt, C.S., Owens, M.M., Crowe, M.L., Carter, N.T., Lynam, D.R., Miller, J.D., 2020. The quandary of covarying: A brief review and empirical examination of covariate use in structural neuroimaging studies on psychological variables. NeuroImage 205, 116225. https://doi.org/10.1016/j.neuroimage.2019.116225

Infante, M.A., Courtney, K.E., Castro, N., Squeglia, L.M., Jacobus, J., 2018. Adolescent brain surface area pre- and post-cannabis and alcohol initiation. J. Stud. Alcohol Drugs 79, 835–843. https://doi.org/10.15288/jsad.2018.79.835

Jacobus, J., Castro, N., Squeglia, L.M., Meloy, M.J., Brumback, T., Huestis, M.A., Tapert, S.F., 2016. Adolescent cortical thickness pre- and post marijuana and alcohol initiation. Neurotoxicol. Teratol. 57, 20–29. https://doi.org/10.1016/j.ntt.2016.09.005

Kann, L., Olsen, E.O., McManus, T., Harris, W.A., Shanklin, S.L., Flint, K.H., Queen, B., Lowry, R., Chyen, D., Whittle, L., Thornton, J., Lim, C., Yamakawa, Y., Brener, N., Zaza, S., 2016. Sexual Identity, Sex of Sexual Contacts, and Health-Related Behaviors Among Students in Grades 9–12 — United States and Selected Sites, 2015. Morb. Mortal. Wkly. Rep. Surveill. Summ. 65, 1–202. https://doi.org/10.15585/mmwr.ss6509a1

Komro, K.A., Maldonado-Molina, M.M., Tobler, A.L., Bonds, J.R., Muller, K.E., 2007. Effects of home access and availability of alcohol on young adolescents’ alcohol use. Addict. Abingdon Engl. 102, 1597–1608. https://doi.org/10.1111/j.1360-0443.2007.01941.x

Koob, G.F., Volkow, N.D., 2010. Neurocircuitry of addiction. Neuropsychopharmacology 35, 217–238. https://doi.org/10.1038/npp.2009.110

Kühn, S., Mascharek, A., Banaschewski, T., Bodke, A., Bromberg, U., Büchel, C., Quinlan, E.B., Desrivieres, S., Flor, H., Grigis, A., Garavan, H., Gowland, P.A., Heinz, A., Ittermann, B., Martinot, J.-L., Nees, F., Papadopoulos Orfanos, D., Paus, T., Poustka, L., Millenet, S., Fröhner, J.H., Smolka, M.N., Walter, H., Whelan, R., Schumann, G., Lindenberger, U., Gallinat, J., IMAGEN Consortium, 2019. Predicting development of adolescent drinking behaviour from whole brain structure at 14 years of age. eLife 8, e44056. https://doi.org/10.7554/eLife.44056

Kuntsche, E., Rossow, I., Engels, R., Kuntsche, S., 2016. Is “age at first drink” a useful concept in alcohol research and prevention? We doubt that. Addict. Abingdon Engl. 111, 957–965. https://doi.org/10.1111/add.12980

Kuntsche, E., Rossow, I., Simons-Morton, B., Bogt, T.T., Kokkevi, A., Godeau, E., 2013. Not early drinking but early drunkenness is a risk factor for problem behaviors among adolescents from 38 European and North American countries. Alcohol. Clin. Exp. Res. 37, 308–314. https://doi.org/10.1111/j.1530-0277.2012.01895.x

Leung, R.K., Toumbourou, J.W., Hemphill, S.A., 2014. The effect of peer influence and selection processes on adolescent alcohol use: A systematic review of longitudinal studies. Health Psychol. Rev. 8, 426–457. https://doi.org/10.1080/17437199.2011.587961

Loeber, R., Farrington, D.P., Stouthamer-Loeber, M., Van Kammen, W.B., 1998. , in: Antisocial Behavior and Mental Health Problems: Explanatory Factors in Childhood and Adolescence. Psychology Press.

Lynam, D.R., Smith, G.T., Whiteside, S.P., Cyders, M.A., 2006. The UPPS-P: Assessing five personality pathways to impulsive behavior. West Lafayette Purdue Univ. 10.

Maechler, M., Rousseeuw, P., Croux, C., Todorov, V., Ruckstuhl, A., Salibian-Barrera, M., Verbeke, T., Koller, M., Conceicao, E.L.T., di Palma, M.A., 2023. robustbase: basic robust statistics R package version 0.99-0.

Maggs, J.L., Patrick, M.E., Feinstein, L., 2008. Childhood and adolescent predictors of alcohol use and problems in adolescence and adulthood in the National Child Development Study. Addiction 103, 7–22. https://doi.org/10.1111/j.1360-0443.2008.02173.x

Maggs, J.L., Staff, J., Patrick, M.E., Wray-Lake, L., 2019. Very early drinking: Event history models predicting alcohol use initiation from age 4 to 11 years. Addict. Behav. 89, 121–127. https://doi.org/10.1016/j.addbeh.2018.09.030

Manuweera, T., Kisner, M.A., Almira, E., Momenan, R., 2022. Alcohol use disorder-associated structural and functional characteristics of the insula. J. Neurosci. Res. 100, 2077–2089. https://doi.org/10.1002/jnr.25113

Marshal, M.P., Friedman, M.S., Stall, R., King, K.M., Miles, J., Gold, M.A., Bukstein, O.G., Morse, J.Q., 2008. Sexual orientation and adolescent substance use: A meta-analysis and methodological review. Addict. Abingdon Engl. 103, 546–556. https://doi.org/10.1111/j.1360-0443.2008.02149.x

Martin, C.S., Winters, K.C., 1998. Diagnosis and assessment of alcohol use disorders among adolescents. Alcohol Health Res. World 22, 95–105.

Meda, S.A., Hawkins, K.A., Dager, A.D., Tennen, H., Khadka, S., Austad, C.S., Wood, R.M., Raskin, S., Fallahi, C.R., Pearlson, G.D., 2018. Longitudinal effects of alcohol consumption on the hippocampus and parahippocampus in college students. Biol. Psychiatry Cogn. Neurosci. Neuroimaging 3, 610–617. https://doi.org/10.1016/j.bpsc.2018.02.006

Meque, I., Dachew, B.A., Maravilla, J.C., Salom, C., Alati, R., 2019. Externalizing and internalizing symptoms in childhood and adolescence and the risk of alcohol use disorders in young adulthood: A meta-analysis of longitudinal studies. Aust. N. Z. J. Psychiatry 53, 965–975. https://doi.org/10.1177/0004867419844308

Miech, R.A., Johnston, L.D., Patrick, M.E., O’Malley, P.M., Bachman, J.G., Schulenberg, J.E., 2023. Monitoring the Future National Survey results on drug use, 1975-2022: Secondary school students. Inst. Soc. Res.

Mills, R., Mann, M.J., Smith, M.L., Kristjansson, A.L., 2021. Parental support and monitoring as associated with adolescent alcohol and tobacco use by gender and age. BMC Public Health 21, 2000. https://doi.org/10.1186/s12889-021-12119-3

Morean, M.E., Kong, G., Camenga, D.R., Cavallo, D.A., Connell, C., Krishnan-Sarin, S., 2014. First drink to first drunk: Age of onset and delay to intoxication are associated with adolescent alcohol use and binge drinking. Alcohol. Clin. Exp. Res. 38, 2615–2621. https://doi.org/10.1111/acer.12526

Morrison, C.N., Byrnes, H.F., Miller, B.A., Wiehe, S.E., Ponicki, W.R., Wiebe, D.J., 2019. Exposure to alcohol outlets, alcohol access, and alcohol consumption among adolescents. Drug Alcohol Depend. 205, 107622. https://doi.org/10.1016/j.drugalcdep.2019.107622

Nagelkerke, N.J.D., 1991. A note on a general definition of the coefficient of determination. Biometrika 78, 691–692. https://doi.org/10.1093/biomet/78.3.691

National Survey on Drug Use and Health, 2022. National Survey on Drug Use and Health (NSDUH) Population Data [WWW Document]. URL https://www.datafiles.samhsa.gov/dataset/national-survey-drug-use-and-health-2022-nsduh-2022-ds0001 (accessed 6.3.24).

O’Brien, J.W., Hill, S.Y., 2017. Neural predictors of substance use disorders in young adulthood. Psychiatry Res. Neuroimaging 268, 22–26. https://doi.org/10.1016/j.pscychresns.2017.08.006

Petersen, A.C., Crockett, L., Richards, M., Boxer, A., 1988. A self-report measure of pubertal status: Reliability, validity, and initial norms. J. Youth Adolesc. 17, 117–133. https://doi.org/10.1007/BF01537962

Pfefferbaum, A., Kwon, D., Brumback, T., Thomson, W.K., Cummins, K., Tapert, S.F., Brown, S.A., Colrain, I.M., Baker, F.C., Prouty, D., De Bellis, M.D., Clark, D.B., Nagel, B.J., Chu, W., Park, S.H., Pohl, K.M., Sullivan, E.V., 2018. Altered brain developmental trajectories in adolescents after initiating drinking. Am. J. Psychiatry 175, 370–380. https://doi.org/10.1176/appi.ajp.2017.17040469

Pfefferbaum, A., Rohlfing, T., Pohl, K.M., Lane, B., Chu, W., Kwon, D., Nolan Nichols, B., Brown, S.A., Tapert, S.F., Cummins, K., Thompson, W.K., Brumback, T., Meloy, M.J., Jernigan, T.L., Dale, A., Colrain, I.M., Baker, F.C., Prouty, D., De Bellis, M.D., Voyvodic, J.T., Clark, D.B., Luna, B., Chung, T., Nagel, B.J., Sullivan, E.V., 2016. Adolescent development of cortical and white matter structure in the NCANDA sample: Role of sex, ethnicity, puberty, and alcohol drinking. Cereb. Cortex N. Y. N 1991 26, 4101–4121. https://doi.org/10.1093/cercor/bhv205

Pilatti, A., Godoy, J.C., Brussino, S.A., Pautassi, R.M., 2013. Patterns of substance use among Argentinean adolescents and analysis of the effect of age at first alcohol use on substance use behaviors. Addict. Behav. 38, 2847–2850. https://doi.org/10.1016/j.addbeh.2013.08.007

R Core Team, 2023. R: A language and environment for statistical computing.

Rane, R.P., de Man, E.F., Kim, J., Görgen, K., Tschorn, M., Rapp, M.A., Banaschewski, T., Bokde, A.L., Desrivieres, S., Flor, H., Grigis, A., Garavan, H., Gowland, P.A., Brühl, R., Martinot, J.-L., Martinot, M.-L.P., Artiges, E., Nees, F., Papadopoulos Orfanos, D., Lemaitre, H., Paus, T., Poustka, L., Fröhner, J., Robinson, L., Smolka, M.N., Winterer, J., Whelan, R., Schumann, G., Walter, H., Heinz, A., Ritter, K., IMAGEN consortium, 2022. Structural differences in adolescent brains can predict alcohol misuse. eLife 11, e77545. https://doi.org/10.7554/eLife.77545

Rice, J.P., Reich, T., Bucholz, K.K., Neuman, R.J., Fishman, R., Rochberg, N., Hesselbrock, V.M., Nurnberger, J.I., Schuckit, M.A., Begleiter, H., 1995. Comparison of direct interview and family history diagnoses of alcohol dependence. Alcohol. Clin. Exp. Res. 19, 1018–1023. https://doi.org/10.1111/j.1530-0277.1995.tb00983.x

Sartor, C.E., Jackson, K.M., McCutcheon, V.V., Duncan, A.E., Grant, J.D., Werner, K.B., Bucholz, K.K., 2016. Progression from first drink, first intoxication, and regular drinking to alcohol use disorder: A comparison of African American and European American youth. Alcohol. Clin. Exp. Res. 40, 1515–1523. https://doi.org/10.1111/acer.13113

Sartor, C.E., Waldron, M., Duncan, A.E., Grant, J.D., McCutcheon, V.V., Nelson, E.C., Madden, P.A.F., Bucholz, K.K., Heath, A.C., 2013. Childhood sexual abuse and early substance use in adolescent girls: the role of familial influences. Addiction 108, 993–1000. https://doi.org/10.1111/add.12115

Schober, P., Vetter, T.R., 2018. Survival analysis and interpretation of time-to-event data: The tortoise and the hare. Anesth. Analg. 127, 792–798. https://doi.org/10.1213/ANE.0000000000003653

Sellers, C.M., McManama O’Brien, K.H., Hernandez, L., Spirito, A., 2018. Adolescent alcohol use: The effects of parental knowledge, peer substance use, and peer tolerance of use. J. Soc. Soc. Work Res. 9, 69–87. https://doi.org/10.1086/695809

Smith, C.L., Cooper, B.R., Miguel, A., Hill, L., Roll, J., McPherson, S., 2021. Predictors of cannabis and tobacco co-use in youth: exploring the mediating role of age at first use in the population assessment of tobacco health (PATH) study. J. Cannabis Res. 3, 16. https://doi.org/10.1186/s42238-021-00072-2

Spilsbury, J.C., Drotar, D., Rosen, C.L., Redline, S., 2007. The Cleveland Adolescent Sleepiness Questionnaire. J. Clin. Sleep Med. JCSM Off. Publ. Am. Acad. Sleep Med. 3, 603–612.

Squeglia, L.M., Ball, T.M., Jacobus, J., Brumback, T., McKenna, B.S., Nguyen-Louie, T.T., Sorg, S.F., Paulus, M.P., Tapert, S.F., 2017. Neural predictors of initiating alcohol use during adolescence. Am. J. Psychiatry 174, 172–185. https://doi.org/10.1176/appi.ajp.2016.15121587

Squeglia, L.M., Cservenka, A., 2017. Adolescence and drug use vulnerability: Findings from neuroimaging. Curr. Opin. Behav. Sci. 13, 164–170. https://doi.org/10.1016/j.cobeha.2016.12.005

Squeglia, L.M., Rinker, D.A., Bartsch, H., Castro, N., Chung, Y., Dale, A.M., Jernigan, T.L., Tapert, S.F., 2014. Brain volume reductions in adolescent heavy drinkers. Dev. Cogn. Neurosci. 9, 117–125. https://doi.org/10.1016/j.dcn.2014.02.005

Tobler, A.L., Komro, K.A., Maldonado-Molina, M.M., 2009. Relationship between neighborhood context, family management practices and alcohol use among urban, multi-ethnic, young adolescents. Prev. Sci. Off. J. Soc. Prev. Res. 10, 313–324. https://doi.org/10.1007/s11121-009-0133-1

Trucco, E.M., 2020. A review of psychosocial factors linked to adolescent substance use. Pharmacol. Biochem. Behav. 196, 172969. https://doi.org/10.1016/j.pbb.2020.172969

Trujillo, C.A., Obando, D., Trujillo, A., 2019. An examination of the association between early initiation of substance use and interrelated multilevel risk and protective factors among adolescents. PLOS ONE 14, e0225384. https://doi.org/10.1371/journal.pone.0225384

Uddin, L.Q., Nomi, J.S., Hebert-Seropian, B., Ghaziri, J., Boucher, O., 2017. Structure and function of the human insula. J. Clin. Neurophysiol. Off. Publ. Am. Electroencephalogr. Soc. 34, 300–306. https://doi.org/10.1097/WNP.0000000000000377

Urošević, S., Collins, P., Muetzel, R., Schissel, A., Lim, K.O., Luciana, M., 2015. Effects of reward sensitivity and regional brain volumes on substance use initiation in adolescence. Soc. Cogn. Affect. Neurosci. 10, 106–113. https://doi.org/10.1093/scan/nsu022

U.S. Census Bureau, 2023a. County Business Patterns Datasets [WWW Document]. URL https://www.census.gov/programs-surveys/cbp/data/datasets.html (accessed 12.20.23).

U.S. Census Bureau, 2023b. American Community Survey (ACS) [WWW Document]. Census.gov. URL https://www.census.gov/programs-surveys/acs (accessed 12.20.23).

U.S. Census Bureau, 2023c. Census Glossary [WWW Document]. Glossary. URL https://www.census.gov/glossary/?term=Unemployed (accessed 12.20.23).

U.S. Census Bureau, 2022. American Community Survey and Puerto Rico Community Survey Design and Methodology. American Community Survey (ACS).

Visser, L., de Winter, A.F., Vollebergh, W.A.M., Verhulst, F.C., Reijneveld, S.A., 2015. Do child’s psychosocial functioning, and parent and family characteristics predict early alcohol use? The TRAILS Study. Eur. J. Public Health 25, 38–43. https://doi.org/10.1093/eurpub/cku072

Warner, L.A., White, H.R., 2003. Longitudinal effects of age at onset and first drinking situations on problem drinking. Subst. Use Misuse 38, 1983–2016. https://doi.org/10.1081/JA-120025123

Whelan, R., Watts, R., Orr, C.A., Althoff, R.R., Artiges, E., Banaschewski, T., Barker, G.J., Bokde, A.L.W., Büchel, C., Carvalho, F.M., Conrod, P.J., Flor, H., Fauth-Bühler, M., Frouin, V., Gallinat, J., Gan, G., Gowland, P., Heinz, A., Ittermann, B., Lawrence, C., Mann, K., Martinot, J.-L., Nees, F., Ortiz, N., Paillère-Martinot, M.-L., Paus, T., Pausova, Z., Rietschel, M., Robbins, T.W., Smolka, M.N., Ströhle, A., Schumann, G., Garavan, H., 2014. Neuropsychosocial profiles of current and future adolescent alcohol misusers. Nature 512, 185–189. https://doi.org/10.1038/nature13402

Zeileis, A., Kleiber, C., Jackman, S., 2008. Regression Models for Count Data in R. J. Stat. Softw. 27.

**Supplement**

**1 Detailed Methods**

***1.1 Measures***

All predictor measures were assessed at the first assessment upon study entry (i.e., baseline). Each measure is described in detail below.

**1.1.1 Youth psychodevelopmental factors.**

*Pubertal development.* Youth completed the Pubertal Developmental Scale (Carskadon and Acebo, 1993; Petersen et al., 1988) until full development was reached to document changes in secondary sex characteristics throughout the duration of the study. Sex-specific changes were assessed, such as body hair, changes in skin, breast growth, and facial hair. Youth rated each characteristic on a 4-point scale (*has not begun, has barely started, is definitely underway, and seems complete*). A single composite score was derived based on the mean of all items.

*Academic and psychological functioning*. Academic performance was assessed based on self-reported grade point average on a 4.0 scale. Academic and career intentions were assessed with a single item in which participants were asked to select their long-term plans from six response options: *1) Finish high school then get a job, 2) Go to college, 3) Go to college and then go to graduate school (or medical school or law school), 4) Enlist in the military, 5) Go to trade school or vocational school, 6) Other*. Based on examinations of data spread, the present analyses recoded responses into three categories of “*Go to college,” “Go to college and then go to graduate school,” and “Other.”*

Internalizing and Externalizing symptoms were assessed with the Youth Self Report (YSR, <18 years old; Achenbach, 1991) or Adult Self Report (ASR, age 18 or older; Achenbach and Rescorla, 2003). Raw scores for each subscale were used in analyses.

*Personality and traits***.** Youth characteristics on the Big-Five personality dimensions were assessed using the brief Ten Item Personality Inventory (Gosling et al., 2003). On a 7-point Likert scale ranging from “*Disagree strongly*” to “*Agree strongly*”, participants indicated the extent to which they agreed each personality trait described them (e.g., “*I see myself as extraverted, enthusiastic*”). Composite scores in five traits were derived by calculating the mean score of items comprising each domain: Agreeableness, Conscientiousness, Emotional Stability, Extraversion, and Openness to Experiences.

Impulsivity traits were assessed with the 20-item Urgency-Premeditation-Perseverance-Sensation Seeking-Positive Urgency (UPPS-P) Impulsive Behavior Scale (Cyders et al., 2007; Lynam et al., 2006). On a 4-point Likert scale ranging from “*Agree strongly*” to “*Disagree strongly*,” youth indicated the extent to which they agree with statements describing various self-characteristics (e.g., “*I finish what I start*”). Composite scores examining five facets of impulsivity were derived by calculating the mean score of items comprising each domain: Negative Urgency, Lack of Premeditation, Lack of Perseverance, Positive Urgency, and Sensation Seeking.

Self-appraisal of executive functioning was assessed using the 80-item Behavior Rating Inventory of Executive Function – Self-Report Version (BRIEF-SR) (Gioia et al., 2002). Youth rated the frequency at which they experienced executive dysfunction over the past six months on a 3-point scale (*Never, Sometimes, Often*). Example items include “*I have a short attention span*” and “*I am impulsive*.” Scores were derived for one summary score (Global Executive Composite), two global composite indices (Behavioral Regulation Index and Metacognition Index) and eight subscales: Inhibitory Control, Flexibility, Emotional Control, Monitoring, Working Memory, Planning, Organization, and Task-Completion. Raw scores were used in analyses.

Expectancies about outcomes following alcohol consumption were assessed with the Alcohol Expectancies Questionnaire (Brown et al., 1987). The 21-item instrument queried youth on the extent to which they agreed with statements related to the impact of alcohol (e.g., “*Alcohol makes people more relaxed and less tense*”) on a 5-point Likert scale ranging from “*disagree strongly*” to “*agree strongly*.” Composite scores assessed positive alcohol expectancies in six domains: Changes in Social Behavior, Increased Arousal, Improved Cognitive and Motor Ability, Relaxation and Tension Reduction, Sexual Enhancement, and Global Positive Change.

*Sleep Patterns***.** Two primary sleep habits metrics were used in the present study’s analyses. Daytime sleepiness was assessed using the 5-item abbreviated Cleveland Adolescent Sleepiness Questionnaire (CASQ; Spilsbury et al., 2007). This metric examined the extent to which youth experienced daytime sleepiness in the past two weeks (e.g., “*I fall asleep in my afternoon activities*”). Circadian pattern was assessed using the 4-item abbreviated Composite Scale of Morningness (Smith et al., 2021) based on the individual’s preferred sleep and wake time, difficulty getting up in the morning, and identification with being a morning or evening type.

**1.1.2 Cognition factors.**

*Neuropsychological functioning.* Neuropsychological functioning was assessed with the computerized Penn Computerized Neurocognitive Battery (Penn CNB; Gur et al., 2010). The present analyses examined youth performance at baseline on 11 tasks: Penn Facial Memory Test (immediate and delayed), Penn Word Memory Test (immediate and delayed), Penn Continuous Performance Test-Number Letter Version, Penn Matrix Analysis Test, Penn Short Visual Object Learning Test (immediate and delayed), Penn Emotion Recognition Test, Penn Conditional Exclusion Task, Penn Measured Emotion Differentiation, and Penn Logical Reasoning. Raw, non-normed, scores (e.g., time in milliseconds) were used for all tasks as age and sex were included as covariates in all analyses. Tasks not included in analyses were omitted due to limited literature suggesting association with adolescent alcohol use (e.g., motor praxis) or introduces additional redundancy in cognitive domains assessed. Non-computerized cognitive performance measures were excluded from analyses due to data incompleteness.

**1.1.3 Family factors.**

*Socioeconomic status.* In addition to race and ethnicity, additional information about the youth’s rearing environment at the family level included parent educational attainment (i.e., socioeconomic status), parent marital status (married vs. other), and current living arrangement (residing with biological parents vs. other).

*Family history*. The Family History Assessment Module (FHAM; Rice et al., 1995) was administered to youth and parent/legal guardian to assess family history of substance use disorder (SUD). Familial history density of alcohol use disorder (AUD) and SUD were calculated as the weighted sum of all first (i.e., biological parents, weighted 0.5) and second-degree relatives (i.e., biological grandparents, weighted 0.25) with lifetime AUD or SUD symptoms, respectively.

*Access to alcohol.* Perceptions on the ease with which youth can gain access to alcohol was assessed using three items from the Access to Substances & Neighborhood Strength Questionnaire, adapted from Komro et al. (2007) and Tobler et al. (Tobler et al., 2009). Each item served as a single-item measure of alcohol access. On a 5-point Likert scale ranging from “*Very difficult*” to “*Very easy*” and “*I don’t know*,” participants indicated the ease of access to obtaining alcohol in the home, from the neighborhood, and a place outside the neighborhood. For ease of interpretation, responses were recoded into a binary scale of “*Easy*” versus “*Not Easy*.”

*Youth-parent relations.* Five measures of parental relationship with youth were examined: parental warmth, solicitation, knowledge, control, and supervision. Parental warmth was assessed using a 6-item instrument (Fletcher et al., 2004) in which youth indicated on a 4-point scale (Never, Sometimes, Usually, Always) the frequency in which parents engaged in behaviors that were perceived as caring, involved, and responsive. Example items included the frequency in which parents offered praise for good grades, encouragement for poor grades, and spend time talking with youth. The mean response of the six items that comprised this scale was calculated to derive a single composite Parental Warmth score.

Parental solicitation was assessed using a 5-item instrument (Fletcher et al., 2004) in which youth indicated on a 3-point scale (*Don’t try to know, Sometimes try to know, Try a lot to know*) the extent to which parents attempted to obtain information regarding their whereabouts, friendships, and activities. Items assessed efforts to obtain this information, regardless of whether parents have actual knowledge in these areas. The question stem asked, “*How much do your parents TRY to know…*” followed by situations such as “*What you do with your free time*” or “*Who your friends are*.” The mean response of the five items that comprised this scale was calculated to derive a single composite Parental Solicitation score.

Parental knowledge examined the accuracy of parent knowledge about youth whereabouts, friendships, and knowledge. Questions were introduced with “*How much do your parents REALLY know…*” about the same items queried for the Parental Solicitation scale. Response options also remained the same. The mean response of the five items that comprised this scale was calculated to derive a single composite Parental Knowledge score (Fletcher et al., 2004).

Parental control, operationalized as the extent to which parents make decisions for youth, was assessed using a 6-item instrument in which youth indicated on a 5-point scale the extent to which they made decisions autonomously from parents. Each item asked, “*How do you make the following decision:*” followed by statements related to bedtime, friends, finances, alcohol use, and dating. Example items included “*How I spend my money*” or “*Whether or not I can drink alcohol*.” Response options included: *I decide this without discussing it with my parents*, *I make the final decision after discussing it with my parents*, *My parents and I make the decision together*, *My parents make the final decision after discussing it with me*, and *My parents decide this without discussing it with me*. The mean response of the six items that comprise this scale was calculated to derive a single composite Parental Control score (Fletcher et al., 2004).

Parental supervision was assessed using a 4-item instrument in which youth indicated on a 3-point scale the frequency with which parents were aware of, and youth informed parents of, their whereabouts outside the home. Example questions included, “*When you are out, do your parent(s) know what time you will be home?*” and “*Do your parent(s) know who you are with when you are away from home?*” The mean response of the four items that comprise this scale was calculated to derive a single composite Parental Supervision score (Loeber et al., 1998; Sartor et al., 2016).

**1.1.4 Peer factors.**

*Peer relations*. Peer relationships were assessed using two single-item measures: “*How many same sex friends do you have*?” and “*How many opposite sex friends do you have?*” Peer alcohol use was assessed using three single-item measures, adapted from Bachman et al. (1981). Youth indicated the estimated number of friends who “*drink alcohol*,” “*get drunk*,” and “*have problems with alcohol*” on a 4-point scale (None, A few, Some, Most, All). Based on examinations of data spread, the present analyses recoded responses into a binary scale of “*None*” and “*A few or more*”.

Romantic partner status was assessed using a single free response item, “*How many different people have you dated or been seeing since you began dating*?” Based on examinations of data spread, the present analyses recoded the item into a binary response indicating zero (i.e., never dated) or non-zero (i.e., has had at least one romantic partner).

**1.1.5 Neighborhood factors.**

*Sociodemographics***.** Neighborhood-level aspects of youth’s rearing environment were assessed based on select sociodemographic metrics from the American Community Survey (ACS; U.S. Census Bureau, 2023b). ACS is a nationwide survey conducted by the United States Census Bureau monthly to estimate population-level housing and sociodemographic characteristics. Monthly data are tabulated and released to the public annually. Methodological details on sample design and selection, data preparation, processing, and dissemination are publicly available through the U.S. Census Bureau (U.S. Census Bureau, 2022).

A total of six ACS sociodemographic metrics were examined in the present study: 1) median household income, 2) percent of population below the national poverty level, 3) percent of adults aged 25 or older with less than 12 years of education (i.e., less than high school diploma or equivalent), 4) percent of adults aged 25 or older with a high school diploma or equivalent, 5) percent of population receiving food stamps or Supplemental Nutrition Assistance Program (SNAP) benefits in the past 12 months 6) percent of population unemployed. Unemployment was defined as civilians aged 16 years and older who are not actively engaged in work, are actively looking for work, and are available to accept a job if offered (U.S. Census Bureau, 2023c). Each ACS sociodemographic metric was obtained for the ZIP Code Tabulation Area (ZCTA) in which youth reported residing at the year of transition into any alcohol use and weekly alcohol use. When geolocation data were not available for the same year of transition, available ZIP code data for the next most proximal year to transition was utilized. Youth geolocation data was available the same year of transition for over 95% of youth in all analyses.

*Alcohol outlet density.* The density, or quantity, of alcohol-related establishments for each youth postal ZIP code at or near the year of transition was calculated using publicly available data from the Census Bureau County Business Pattern (CBP; U.S. Census Bureau, 2023a). The same methodology to assess geolocation data by year of transition for ACS sociodemographic metrics was applied for CBP data.

The North American Industry Classification System (NAICS) is a classification code system used by Federal statistical agencies to classify business establishments. For the present study, businesses with the following NAICS code labels were used to quantify the number of alcohol-related establishments by ZIP code: 1) *Beer, Wine, and Distilled Alcoholic Beverage Merchant Wholesalers*, 2) *Beer and Ale Merchant Wholesalers*, 3) *Wine and Distilled Alcoholic Beverage Merchant Wholesalers*, 4) *Beer, Wine, and Liquor Stores*, and 5) *Drinking Places (Alcoholic Beverages)*. Three metrics of alcohol outlet density were derived for each zip code: 1) the number of beer, wine, and liquor stores, 2) the number of drinking places, and 3) the total number of alcohol-related establishments. The number of alcohol-related wholesalers was not calculated as an independent metric due to the low number of establishments for most ZIP codes examined in the present study.

***1.2 MRI Literature Search***

**Table 4**

MRI regions selected as predictors in Part I analyses.

| **Original study** | **Laterality** | **Region found in original study to be predictive of adolescent alcohol use** | **Volume type examined in original study** | | | **Corresponding FreeSurfer NCANDA region examined in present study** | |
| --- | --- | --- | --- | --- | --- | --- | --- |
| **Frontal regions** | | | | | | |  |
| Baranger et al. (2020); Brumback et al. (2016); Squeglia et al. (2017); Whelan et al. (2014) 3/1/24 2:37:00 PM | Right | Dorsolateral prefrontal cortex | | GMV | Rostral middle frontal GMV; Superior frontal GMV | | |
| Cheetham et al. (2014); Squeglia et al. (2014) | Left | Anterior cingulate cortex | | GMV | Caudal anterior cingulate GMV; Rostral anterior cingulate GMV ^a^ | | |
| Hatoum et al. (2021) | Left | Frontal pole | | GMV | Frontal pole GMV | | |
| Infante et al. (2018) | Left | Medial orbitofrontal cortex | | SA | Medial orbitofrontal SA ^c^ | | |
| Infante et al. (2018) | Right | Medial orbitofrontal cortex | | SA | Medial orbitofrontal SA ^c^ | | |
| Jacobus et al. (2016) | Left | superior frontal gyrus | | Cortical thickness | Superior frontal thickness | | |
| O'Brien et al. (2017) | Right | Orbitofrontal cortex | | GMV | Lateral orbitofrontal GMV; Medial orbitofrontal GMV ^b^ | | |
| Rane et al. (2022) | Left | Pars orbitalis | | SA | Parsorbitalis SA | | |
| Rane et al. (2022) | Left | Rostral middle fontal gyrus | | Thickness | Rostral middle frontal thickness | | |
| Squeglia et al. (2017) | Right | Frontal pole | | Cortical thickness | Frontal pole thickness | | |
| Squeglia et al. (2017) | Right | Pars orbitalis | | Cortical thickness | Parsorbitalis thickness | | |
| Whelan et al. (2014) | Right | Precentral gyrus | | Volume | Precentral GMV | | |
| Whelan et al. (2014) | Left | Superior frontal gyrus | | Volume | Superior frontal GMV | | |
| **Parietal regions** | | | | | | |  |
| Hatoum et al. (2021); Jacobus et al. (2016) | Right | Supramarginal gyrus | Cortical thickness | | | Supramarginal thickness | |
| Rane et al. (2022) | Right | Cuneus cortex | Thickness | | | Cuneus thickness | |
| Rane et al. (2022) | Right | Inferior parietal cortex | Cortical thickness | | | Inferior parietal thickness | |
| Rane et al. (2022) | Left | Pericalcarine cortex | Thickness | | | Pericalcarine thickness | |
| Rane et al. (2022) | Right | Pericalcarine cortex | Thickness | | | Pericalcarine thickness | |
| Squeglia et al. (2017) | Right | Precuneus | Cortical thickness | | | Precuneus thickness | |
| Squeglia et al. (2017) | Right | Superior parietal | Cortical thickness | | | Superior parietal thickness | |
| Squeglia et al. (2017) | Left | Supramarginal gyrus | Cortical thickness | | | Supramarginal thickness | |
| **Temporal regions** | | | | | | |  |
| Rane et al. (2022) | Left | Temporal pole | Volume | | | Temporal pole GMV | |
| Squeglia et al. (2017) | Right | Temporal pole | Cortical thickness | | | Temporal pole thickness | |
| Squeglia et al. (2017) | Left | Transverse temporal cortex | Cortical thickness | | | Transverse temporal thickness | |
| **Occipital regions** | | | | | | |  |
| Rane et al. (2022) | Left | Lateral occipital cortex | Thickness | | | Lateral occipital thickness | |
| **Insular regions** | | | | | | |  |
| Baranger et al. (2020) | Right | Insula | GMV | | | Insula GMV | |
| Infante et al. (2018) | Right | Insula | SA | | | Insula SA ^c^ | |
| **Limbic regions** | | | | | | |  |
| O'Brien et al. (2017) | Right | Amygdala | Volume | | | Amygdala volume | |
| Rane et al. (2022) | Right | Parahippocampal gyrus | SA | | | Parahippocampal SA | |
| Urosevic et al. (2015) | Left | Nucleus accumbens | Volume | | | Accumbens volume | |
| **Other/subcortical regions** | | | | | | |  |
| Rane et al. (2022) | Left | Paracentral lobule | Thickness | | | Paracentral thickness | |
| Rane et al. (2022); Kuhn et al. (2019) 3/1/24 2:37:00 PM | Right | Cerebellar Cortex | Volume | | | Cerebellar volume | |
| Rane et al. (2022) | Right | Globus pallidum | Volume | | | Pallidum volume | |

Literature support for the 31 structural MRI regions selected for examination in the current study are shown in this table. Regions were selected based on those previously found to be prospectively predictive of adolescent alcohol use. See Honarvar et al. (2023) for review. Only studies and regions identified utilizing structural MRI were examined; regions identified in other neuroimaging modalities (e.g., functional MRI, functional connectivity) were not included among candidate predictors. Regions that were found to be associated with, but not predictive of, adolescent alcohol use in cross-sectional studies were also not included. In addition to the above listed, Pfefferbaum et al. (2018) found that heavier drinkers have smaller parahippocampal and middle temporal volume at baseline, but did not survive multiple comparisons correction.

^a^ Gray matter volume for the caudal and rostral anterior cingulate regions were summed to derive a single anterior cingulate volume to maintain consistency with the region identified by Cheetham et al. (2014)

^b^ Gray matter volume for the lateral and medial orbitofrontal gray matter volume were summed to derive a single right orbitofrontal gray matter volume to maintain consistency with the region identified by O’Brien et al. (2017)

^c^ Surface area for these regions were not included in analyses, as thickness and volume information in the same areas were already examined.

*Note*: GMV: Gray matter volume; SA: Surface area

**2 Results**

**Table 5**

Part 1 Cox Proportional Hazards Model Predicting Age of First Drinking Onset: Full Model Results.

| **Predictors that emerged from best subsets selection** | **HR** | **95% CI LB** | **95% CI UB** | **Nominal *p*-value** | **FDR adjusted**  ***p-*value** |
| --- | --- | --- | --- | --- | --- |
| **Model 1: Self-report** | | | | | |
| AEQ Changes in Social Behavior | 1.63 | 1.28 | 2.06 | <0.0001 | 0.0001 |
| AEQ Increased Arousal | 0.84 | 0.69 | 1.01 | 0.07 | 0.09 |
| AEQ Relaxation and Tension Reduction | 1.28 | 1.08 | 1.51 | 0.004 | 0.02 |
| TIPI Conscientiousness | 1.15 | 1.01 | 1.31 | 0.03 | 0.06 |
| TIPI Openness to Experiences | 1.22 | 1.02 | 1.46 | 0.03 | 0.06 |
| UPPS Negative Urgency | 1.18 | 1.02 | 1.37 | 0.03 | 0.06 |
| UPPS Lack of Premeditation | 1.16 | 1.00 | 1.34 | 0.05 | 0.08 |
| UPPS Sensation Seeking | 1.29 | 1.11 | 1.49 | 0.001 | 0.01 |
| Career intentions [ref: Attend college]: Attend graduate school | 1.18 | 0.97 | 1.44 | 0.10 | 0.12 |
| Career intentions [ref: Attend college]: Not attend college | 0.64 | 0.40 | 1.03 | 0.06 | 0.09 |
| Age at study entry | 0.57 | 0.40 | 0.82 | 0.003 | 0.01 |
| **Model 2: Cognition** | | | | | |
| Penn Emotion Recognition Test TC | 1.13 | 0.99 | 1.28 | 0.06 | 0.09 |
| Penn Matrix Analysis Test TC | 0.84 | 0.70 | 1.01 | 0.06 | 0.08 |
| Penn Logical Reasoning TC | 1.21 | 1.03 | 1.43 | 0.02 | 0.05 |
| Penn Short Visual Object Learning Test TC | 1.14 | 0.96 | 1.34 | 0.13 | 0.14 |
| Penn Delayed Short Visual Object Learning Test TC | 0.87 | 0.75 | 1.01 | 0.06 | 0.08 |
| Age at study entry | 0.61 | 0.42 | 0.87 | 0.01 | 0.02 |
| **Model 3: Brain structure** | | | | | |
| R pars orbitalis surface area | 1.11 | 0.97 | 1.26 | 0.12 | 0.14 |
| R frontal pole thickness | 0.86 | 0.75 | 0.99 | 0.04 | 0.07 |
| R insula gray volume | 1.20 | 1.05 | 1.39 | 0.01 | 0.03 |
| R parahippocampal surface area | 0.83 | 0.74 | 0.93 | 0.0001 | 0.01 |
| R pallidum volume | 1.13 | 1.00 | 1.27 | 0.05 | 0.08 |
| Age at study entry | 0.66 | 0.46 | 0.95 | 0.03 | 0.06 |
| **Model 4: Family** | | | | | |
| Family history of alcohol problems | 1.11 | 0.97 | 1.27 | 0.13 | 0.14 |
| Parental control | 0.72 | 0.60 | 0.86 | 0.0003 | 0.003 |
| Parental knowledge | 0.73 | 0.60 | 0.88 | 0.0009 | 0.01 |
| Parental solicitation | 1.68 | 1.39 | 2.02 | <0.0001 | 0.0001 |
| Parental warmth | 1.11 | 0.98 | 1.27 | 0.10 | 0.12 |
| Ease of access to alcohol at home  [ref: Not easy]: Easy | 1.52 | 1.23 | 1.88 | <0.0001 | 0.0001 |
| Ease of access to alcohol in neighborhood [ref: Not Easy]: Easy | 1.37 | 1.05 | 1.80 | 0.02 | 0.05 |
| Age at study entry | 0.52 | 0.36 | 0.75 | 0.0005 | 0.0004 |
| **Model 5: Peers** | | | | | |
| Have friends who drink | 1.57 | 1.23 | 1.98 | 0.0002 | 0.002 |
| Number of same-sex friends | 1.08 | 1.02 | 1.15 | 0.01 | 0.03 |
| Have dated | 1.30 | 1.06 | 1.59 | 0.01 | 0.04 |
| Age at study entry | 0.55 | 0.38 | 0.80 | 0.002 | 0.01 |
| **Model 6: Neighborhood ZIP code** | | | | | |
| ACS ZIP code-based % high school graduation rate | 0.81 | 0.66 | 1.01 | 0.002 | 0.01 |
| ACS ZIP code-based median household income | 0.96 | 0.81 | 1.15 | 0.66 | 0.66 |
| ACS ZIP code-based unemployment rate | 1.42 | 1.25 | 1.60 | <0.0001 | 0.0001 |
| Age at study entry | 0.67 | 0.47 | 0.96 | 0.03 | 0.06 |

Six Cox Proportional Hazard Models predicting age of first drinking onset were computed, one for each domain influencing adolescent drinking behavior: youth self-report, cognition, brain structure, family, peers, and community (Part 1: Best subset of baseline precursors to alcohol use onset). Multiple comparisons correction was applied using a false discovery rate (FDR) of *q* = 0.05. Predictors with nominal (uncorrected) and adjusted *p-*values <0.05 are highlighted in grey.

Predictors were derived from best subsets selection, only those predictors that emerged in the best-fitting model based on the lowest corrected Akaike information Criterion (AICC). All models within 2 AICC units of the best fitting model may be obtained by contacting the study authors.

*Note:*

HR: Hazard ratio

95% CI LB: Hazard ratio 95% Confidence Interval Lower Bound

95% CI UB: Hazard ratio 95% Confidence Interval Upper Bound

Youth self-report factors: AEQ: Alcohol Expectancy Questionnaire; TIPI: Ten Item Personality Inventory; UPPS: Urgency, Premeditation, Perseverance, Sensation Seeking Scale.

Youth cognition factors: TC: total correct

Youth brain structure factors: L: Left; R: Right.

Family factors: AUD: Alcohol Use Disorder; SUD: Substance Use Disorder.

Neighborhood ZIP code factors: U.S. Census Bureau American Community Survey.

**Table 6**

Part 1 Cox Proportional Hazards Model Predicting Age of Regular Drinking Onset: Full Model Results.

| **Predictors that emerged from best subsets selection** | **HR** | **95% CI LB** | **95% CI UB** | **Nominal *p*-value** | **FDR adjusted**  ***p-*value** |
| --- | --- | --- | --- | --- | --- |
| **Model 1: Youth self-report** | | | | | |
| AEQ Changes in Social Behavior | 1.70 | 1.24 | 2.34 | 0.001 | 0.01 |
| AEQ Improved Cognitive and Motor Ability | 0.77 | 0.58 | 1.02 | 0.07 | 0.09 |
| TIPI Agreeableness | 0.79 | 0.63 | 0.98 | 0.03 | 0.06 |
| TIPI Openness to Experiences | 1.28 | 1.02 | 1.61 | 0.03 | 0.06 |
| UPPS Sensation Seeking | 1.38 | 1.14 | 1.69 | 0.001 | 0.01 |
| Grade point average | 1.28 | 1.06 | 1.56 | 0.01 | 0.04 |
| YSR/ASR Externalizing Behaviors | 0.83 | 0.67 | 1.02 | 0.07 | 0.09 |
| YSR/ASR Internalizing Behaviors | 1.17 | 0.98 | 1.41 | 0.09 | 0.11 |
| Age at study entry | 0.66 | 0.42 | 1.03 | 0.07 | 0.09 |
| **Model 2: Cognition** | | | | | |
| Penn Facial Memory Test TC | 1.22 | 1.02 | 1.46 | 0.03 | 0.06 |
| Penn Conditional Exclusion Task Accuracy | 1.32 | 1.09 | 1.61 | 0.01 | 0.02 |
| Penn Matrix Analysis Test TC | 0.79 | 0.62 | 0.99 | 0.04 | 0.07 |
| Penn Logical Reasoning TC | 1.12 | 0.91 | 1.38 | 0.27 | 0.28 |
| Penn Continuous Performance Test TP | 1.11 | 0.99 | 1.25 | 0.07 | 0.09 |
| Age at study entry | 0.64 | 0.41 | 1.01 | 0.06 | 0.08 |
| **Model 3: Brain structure** | | | | | |
| R lateral occipital gyrus thickness | 1.14 | 0.95 | 1.38 | 0.16 | 0.17 |
| R transverse temporal gyrus thickness | 0.90 | 0.76 | 1.06 | 0.21 | 0.22 |
| Age at study entry | 0.88 | 0.56 | 1.39 | 0.58 | 0.58 |
| R insula gray volume | 1.32 | 1.11 | 1.57 | 0.002 | 0.01 |
| R parahippocampal surface area | 0.83 | 0.72 | 0.95 | 0.01 | 0.03 |
| R pars orbitalis thickness | 0.87 | 0.74 | 1.02 | 0.09 | 0.11 |
| R precentral gray volume | 1.19 | 1.00 | 1.42 | 0.05 | 0.08 |
| **Model 4: Family** | | | | | |
| Parent education level | 1.11 | 1.00 | 1.24 | 0.05 | 0.08 |
| Parental control | 0.79 | 0.64 | 0.99 | 0.04 | 0.07 |
| Parental solicitation | 1.80 | 1.43 | 2.27 | <0.0001 | 0.0001 |
| Parental supervision | 1.10 | 0.99 | 1.22 | 0.07 | 0.09 |
| Race [ref: White]: Non-White | 0.71 | 0.53 | 0.94 | 0.02 | 0.047 |
| Ease of access to alcohol at home  [ref: Not easy]: Easy | 1.75 | 1.36 | 2.26 | <0.0001 | 0.0001 |
| **Model 5: Peers** | | | | | |
| Age at study entry | 0.58 | 0.36 | 0.93 | 0.02 | 0.05 |
| Have friends who drink | 1.48 | 1.08 | 2.04 | 0.02 | 0.04 |
| Have friends who have problems with alcohol | 1.46 | 0.99 | 2.16 | 0.05 | 0.08 |
| Number of same-sex friends | 1.09 | 1.00 | 1.19 | 0.06 | 0.08 |
| Have dated | 1.28 | 1.00 | 1.64 | 0.05 | 0.08 |
| **Model 6: Neighborhood ZIP code** | | | | | |
| ACS Zip code % high school graduation rate | 0.80 | 0.61 | 1.06 | 0.09 | 0.11 |
| ACS Zip code median household income | 1.16 | 0.94 | 1.42 | 0.16 | 0.17 |
| ACS Zip code unemployment rate | 1.25 | 1.07 | 1.47 | 0.01 | 0.02 |
| Age at study entry | 0.65 | 0.41 | 1.03 | 0.07 | 0.09 |

Six Cox Proportional Hazard Models predicting age of regular (i.e., weekly) drinking onset were computed, one for each factor influencing adolescent drinking behavior: youth self-report, cognition, brain structure, family, peers, and community (Part 1: Best subset of baseline precursors to alcohol use onset). Multiple comparisons correction was applied using a False Discovery Rate (FDR) of *q* = 0.05. Predictors with nominal (uncorrected) and adjusted *p-*values <0.05 are highlighted in grey. Only predictors of interest (i.e., non-confounder variables) with adjusted *p*-value <0.05 were interpreted.

Predictors were derived from best subsets selection, only those predictors that emerged in the best-fitting model based on the lowest corrected Akaike information Criterion (AICC). All models within 2 AICC units of the best fitting model may be obtained by contacting the study authors.

HR: Hazard ratio

95% CI LB: Hazard ratio 95% Confidence Interval Lower Bound

95% CI UB: Hazard ratio 95% Confidence Interval Upper Bound

Youth self-report factors: AEQ: Alcohol Expectancy Questionnaire; SHQ: Sleep Habits Questionnaire; TIPI: Ten Item Personality Inventory; UPPS: Urgency, Premeditation, Perseverance, Sensation Seeking Scale.

Youth cognition factors: TC: total correct; TP: true positives.

Neighborhood ZIP code factors: U.S. Census Bureau American Community Survey.

Tables 4 – 7 show full model results of robust linear regressions predicting age of binge drinking onset and age of regular binge drinking onset (Part 2: Prospective consequences of early alcohol use onset). The primary predictor of interest is indicated in bold. For each model, the reference groups were: male sex, White race, non-Hispanic ethnicity. Corrected *p*-values indicate *p*-values following Bonferroni correction calculated as 0.05 divided by the number of predictors of interest tested across the six models.

**Table 7**

Part 2 Model Results of Robust Linear Regressions: Age of First Drinking Onset Predicting Age of Binge Drinking Onset.

|  | β | *CI* | Nominal  *p*-value |
| --- | --- | --- | --- |
| **Age of first drinking onset** | **0.85** | **0.77 – 0.93** | **<0.0001** |
| Sex: Female | -0.12 | -0.33 – 0.09 | 0.25 |
| Family history density of alcohol problems | -0.15 | -0.29 – -0.02 | **0.02** |
| Parent educational attainment | -0.03 | -0.08 – 0.02 | 0.30 |
| Race: Non-White | 0.21 | -0.07 – 0.48 | 0.14 |
| Ethnicity: Hispanic | -0.20 | -0.49 – 0.09 | 0.17 |
| Age at study onset | 0.07 | 0.01 – 0.14 | **0.03** |
| N = 367  Adjusted R^2^ = 0.78 |  |  |  |

**Table 8**

Part 2 model Results of Robust Linear Regressions: Age of First Drinking Onset Predicting Age of Regular Binge Drinking Onset.

|  | β | *CI* | Nominal  *p*-value |
| --- | --- | --- | --- |
| **Age of first drinking onset** | **0.49** | **0.30 – 0.67** | **<0.0001** |
| Sex: Female | -0.10 | -0.60 – 0.40 | 0.69 |
| Family history density of alcohol problems | 0.21 | -0.10 – 0.52 | 0.19 |
| Parent educational attainment | -0.00 | -0.21 – 0.21 | 0.99 |
| Race: Non-White | 0.17 | -0.44 – 0.78 | 0.58 |
| Ethnicity: Hispanic | -1.19 | -1.91 – -0.48 | **0.001** |
| Age at study onset | 0.13 | -0.05 – 0.31 | 0.17 |
| N = 114  Adjusted R^2^ = 0.38 |  |  |  |

**Table 9**

Part 2 Model Results of Robust Linear Regressions: Age of Regular Drinking Onset Predicting Age of Binge Drinking Onset.

|  | β | *CI* | Nominal  *p*-value |
| --- | --- | --- | --- |
| **Age of regular drinking onset** | **0.50** | **0.37 – 0.64** | **<0.0001** |
| Sex: Female | -0.04 | -0.40 – 0.31 | 0.81 |
| Family history density of alcohol problems | -0.31 | -0.59 – -0.02 | **0.03** |
| Parent educational attainment | -0.02 | -0.12 – 0.07 | 0.64 |
| Race: Non-White | 0.33 | -0.10 – 0.77 | 0.13 |
| Ethnicity: Hispanic | -0.08 | -0.68 – 0.53 | 0.80 |
| Age at study onset | 0.32 | 0.23 – 0.42 | **<0.001** |
| N = 273  Adjusted R^2^ = 0.51 |  |  |  |

**Table 10**

Part 2 Model Results of Robust Linear Regressions: Age of Regular Drinking Onset Predicting Age of Weekly Binge Drinking Onset.

|  | β | *CI* | Nominal  *p*-value |
| --- | --- | --- | --- |
| **Age of regular drinking onset** | **0.97** | **0.91 – 1.04** | **<0.0001** |
| Sex: Female | 0.06 | -0.19 – 0.30 | 0.66 |
| Family history density of alcohol problems | 0.03 | -0.07 – 0.14 | 0.53 |
| Parent educational attainment | 0.05 | -0.04 – 0.14 | 0.28 |
| Race: Non-White | -0.04 | -0.22 – 0.14 | 0.67 |
| Ethnicity: Hispanic | -0.18 | -0.43 – 0.08 | 0.18 |
| Age at study onset | -0.00 | -0.05 – 0.05 | 0.94 |
| N = 114  Adjusted R^2^ = 0.94 |  |  |  |

Tables 8 – 9 show full model results of zero-inflated Poisson models predicting number of lifetime withdrawal symptoms (Part 2: Prospective consequences of early alcohol use onset). The primary predictor of interest is indicated in bold. For each model, the reference groups were: male sex, White race, non-Hispanic ethnicity. Corrected *p*-values indicated *p*-values following multiple comparisons correction with a false discovery rate of *q* = 0.05.

**Table 11**

Part 2 Model Results of Zero-Inflated Poisson Regression Predicting Lifetime Withdrawal Symptoms.

|  | Incidence rate ratio | 95% CI | Nominal  *p*-value |
| --- | --- | --- | --- |
| **Count Poisson model** |  |  |  |
| **Age of first drinking onset** | **0.99** | **0.93 – 1.06** | **0.78** |
| Sex: Female | 1.03 | 0.88 – 1.21 | 0.73 |
| Family history density of alcohol problems | 1.04 | 0.94 – 1.16 | 0.45 |
| Parent educational attainment | 0.99 | 0.96 – 1.03 | 0.75 |
| Race: Non-White | 1.00 | 0.82 – 1.23 | 0.99 |
| Ethnicity: Hispanic | 0.75 | 0.50 – 1.12 | 0.16 |
| Age at study onset | 1.01 | 0.95 – 1.07 | 0.82 |
| **Zero-inflated logit model** |  |  |  |
| **Age of first drinking onset** | **1.62** | **1.40 – 1.88** | **<0.0001** |
| Sex: Female | 0.94 | 0.62 – 1.44 | 0.78 |
| Family history density of alcohol problems | 0.89 | 0.65 – 1.21 | 0.45 |
| Parent educational attainment | 0.96 | 0.87 – 1.06 | 0.39 |
| Race: Non-White | 0.56 | 0.34 – 0.92 | 0.02 |
| Ethnicity: Hispanic | 2.67 | 1.20 – 5.96 | 0.02 |
| Age at study onset | 0.73 | 0.64 – 0.83 | <0.001 |
| N = 454  Adjusted R^2^ = 0.27 |  |  |  |

**Table 9**

Part 2 Model Results of Zero-Inflated Poisson Regression Predicting Lifetime Withdrawal Symptoms.

|  | Incidence rate ratio | 95% CI | Nominal  *p*-value |
| --- | --- | --- | --- |
| **Count Poisson model** |  |  |  |
| **Age of first drinking onset** | **1.00** | **0.95 – 1.06** | **0.97** |
| Sex: Female | 0.99 | 0.84 – 1.17 | 0.91 |
| Family history density of alcohol problems | 1.02 | 0.92 – 1.14 | 0.66 |
| Parent educational attainment | 1.01 | 0.97 – 1.05 | 0.70 |
| Race: Non-White | 0.98 | 0.79 – 1.22 | 0.88 |
| Ethnicity: Hispanic | 0.65 | 0.41 – 1.02 | 0.06 |
| Age at study onset | 0.98 | 0.94 – 1.03 | 0.54 |
| **Zero-inflated logit model** |  |  |  |
| **Age of first drinking onset** | **1.29** | **1.08 – 1.53** | **0.004** |
| Sex: Female | 0.80 | 0.49 – 1.33 | 0.40 |
| Family history density of alcohol problems | 0.78 | 0.55 – 1.13 | 0.19 |
| Parent educational attainment | 1.02 | 0.90 – 1.15 | 0.76 |
| Race: Non-White | 0.70 | 0.38 – 1.27 | 0.24 |
| Ethnicity: Hispanic | 2.89 | 1.18 – 7.08 | **0.02** |
| Age at study onset | 0.83 | 0.71 – 0.96 | **0.02** |
| N = 287  Adjusted R^2^ = 0.17 |  |  |  |

**Figures**

**Supplemental Fig. 1.** Time between transition from first drink to regular drinking and first binge episode to regular binge drinking (N = 538). The left panel displays the number of years between first binge drinking episode to regular binge drinking onset and the number of participants at each duration; the right panel displays the number of years between first drink to regular drinking onset and the number of participants at each duration. A duration of zero indicate that participants transitioned the same year.

**Supplemental Fig 2.** Age at first and regular drinking onset (N = 538). The left panel displays the number of participants at each age of first drink; the right panel displays the number of participants at each age at regular drinking onset.

**References: Supplement**

Abu-Bader, S.H., 2010. Advanced and multivariate statistical methods for social science research. Oxford University Press.

Achenbach, T.M., 1991. Manual for the youth self-report and 1991 profile. University of Vermont Department of Psychiatry.

Achenbach, T.M., Rescorla, L.A., 2003. Manual for the ASEBA adult forms & profiles. Burlington, VT: University of Vermont, Research Center for Children, Youth and Families, Burlington, VT.

Alexander, J.D., Freis, S.M., Zellers, S.M., Corley, R., Ledbetter, A., Schneider, R.K., Phelan, C., Subramonyam, H., Frieser, M., Rea-Sandin, G., Stocker, M.E., Vernier, H., Jiang, M., Luo, Y., Zhao, Q., Rhea, S.A., Hewitt, J., Luciana, M., McGue, M., Wilson, S., Resnick, P., Friedman, N.P., Vrieze, S.I., 2023. Evaluating longitudinal relationships between parental monitoring and substance use in a multi-year, intensive longitudinal study of 670 adolescent twins. Front. Psychiatry 14. https://doi.org/10.3389/fpsyt.2023.1149079

Armenta, B.E., Sittner, K.J., Whitbeck, L.B., 2016. Predicting the Onset of Alcohol Use and the Development of Alcohol Use Disorder Among Indigenous Adolescents. Child Dev. 87, 870–882. https://doi.org/10.1111/cdev.12506

Bachman, J.G., 1981. Monitoring the Future: Questionnaire Responses from the Nation’s High School Seniors, 1980. ERIC.

Baranger, D.A.A., Demers, C.H., Elsayed, N.M., Knodt, A.R., Radtke, S.R., Desmarais, A., Few, L.R., Agrawal, A., Heath, A.C., Barch, D.M., Squeglia, L.M., Williamson, D.E., Hariri, A.R., Bogdan, R., 2020. Convergent evidence for predispositional effects of brain gray matter volume on alcohol consumption. Biol. Psychiatry 87, 645–655. https://doi.org/10.1016/j.biopsych.2019.08.029

Bekman, N.M., Cummins, K., Brown, S.A., 2010. Affective and Personality Risk and Cognitive Mediators of Initial Adolescent Alcohol Use. J. Stud. Alcohol Drugs 71, 570–580. https://doi.org/10.15288/jsad.2010.71.570

Bertsimas, D., King, A., Mazumder, R., 2016. Best subset selection via a modern optimization lens. Ann. Stat. 44, 813–852. https://doi.org/10.1214/15-AOS1388

Bray, J.H., Gallegos, M.I., Cain, M.K., Zaring-Hinkle, B., 2022. Parental monitoring, family conflict, and adolescent alcohol use: A longitudinal latent class analysis. J. Fam. Psychol. 36, 1154–1160. https://doi.org/10.1037/fam0001019

Bronfenbrenner, U., 1974. Developmental Research, Public Policy, and the Ecology of Childhood. Child Dev. 45, 1–5. https://doi.org/10.2307/1127743

Bronfenbrenner, U., Ceci, S.J., 1994. Nature-nuture reconceptualized in developmental perspective: A bioecological model. Psychol. Rev. 101, 568–586. https://doi.org/10.1037/0033-295X.101.4.568

Brown, S.A., Brumback, T., Tomlinson, K., Cummins, K., Thompson, W.K., Nagel, B.J., De Bellis, M.D., Hooper, S.R., Clark, D.B., Chung, T., Hasler, B.P., Colrain, I.M., Baker, F.C., Prouty, D., Pfefferbaum, A., Sullivan, E.V., Pohl, K.M., Rohlfing, T., Nichols, B.N., Chu, W., Tapert, S.F., 2015. The National Consortium on Alcohol and NeuroDevelopment in Adolescence (NCANDA): A Multisite Study of Adolescent Development and Substance Use. J. Stud. Alcohol Drugs 76, 895–908. https://doi.org/10.15288/jsad.2015.76.895

Brown, S.A., Christiansen, B.A., Goldman, M.S., 1987. The Alcohol Expectancy Questionnaire: an instrument for the assessment of adolescent and adult alcohol expectancies. J. Stud. Alcohol 48, 483–491. https://doi.org/10.15288/jsa.1987.48.483

Brown, S.A., Myers, M.G., Lippke, L., Tapert, S.F., Stewart, D.G., Vik, P.W., 1998. Psychometric evaluation of the Customary Drinking and Drug Use Record (CDDR): a measure of adolescent alcohol and drug involvement. J. Stud. Alcohol 59, 427–438. https://doi.org/10.15288/jsa.1998.59.427

Brumback, T.Y., Worley, M., Nguyen-Louie, T.T., Squeglia, L.M., Jacobus, J., Tapert, S.F., 2016. Neural predictors of alcohol use and psychopathology symptoms in adolescents. Dev. Psychopathol. 28, 1209–1216. https://doi.org/10.1017/S0954579416000766

Calcagno, V., de Mazancourt, C., 2010. glmulti: An R Package for Easy Automated Model Selection with (Generalized) Linear Models. Journal of Statistical Software 34, 1–29. https://doi.org/10.18637/jss.v034.i12

Campbell, E.J., Lawrence, A.J., 2021. It’s more than just interoception: The insular cortex involvement in alcohol use disorder. J. Neurochem. 157, 1644–1651. https://doi.org/10.1111/jnc.15310

Carskadon, M.A., Acebo, C., 1993. A self-administered rating scale for pubertal development. J. Adolesc. Health Off. Publ. Soc. Adolesc. Med. 14, 190–195. https://doi.org/10.1016/1054-139x(93)90004-9

Cheetham, A., Allen, N.B., Whittle, S., Simmons, J., Yücel, M., Lubman, D.I., 2014. Volumetric differences in the anterior cingulate cortex prospectively predict alcohol-related problems in adolescence. Psychopharmacology (Berl.) 231, 1731–1742. https://doi.org/10.1007/s00213-014-3483-8

Chen, M.-J., Grube, J.W., Gruenewald, P.J., 2010. Community alcohol outlet density and underage drinking. Addict. Abingdon Engl. 105, 270–278. https://doi.org/10.1111/j.1360-0443.2009.02772.x

Clark, T.G., Bradburn, M.J., Love, S.B., Altman, D.G., 2003. Survival analysis part I: Basic concepts and first analyses. Br. J. Cancer 89, 232–238. https://doi.org/10.1038/sj.bjc.6601118

Connor, J.P., Weier, M., Hall, W.D., 2019. The Age of Onset of Alcohol Use Disorders, in: de Girolamo, G., McGorry, P.D., Sartorius, N. (Eds.), Age of Onset of Mental Disorders: Etiopathogenetic and Treatment Implications. Springer International Publishing, Cham, pp. 169–182. https://doi.org/10.1007/978-3-319-72619-9_9

Cyders, M.A., Smith, G.T., Spillane, N.S., Fischer, S., Annus, A.M., Peterson, C., 2007. Integration of impulsivity and positive mood to predict risky behavior: Development and validation of a measure of positive urgency. Psychol. Assess. 19, 107–118. https://doi.org/10.1037/1040-3590.19.1.107

Dawson, D.A., Goldstein, R.B., Patricia Chou, S., June Ruan, W., Grant, B.F., 2008. Age at first drink and the first incidence of adult-onset DSM-IV alcohol use disorders. Alcohol. Clin. Exp. Res. 32, 2149–2160. https://doi.org/10.1111/j.1530-0277.2008.00806.x

DeWit, D.J., Adlaf, E.M., Offord, D.R., Ogborne, A.C., 2000. Age at first alcohol use: A risk factor for the development of alcohol disorders. Am. J. Psychiatry 157, 745–750. https://doi.org/10.1176/appi.ajp.157.5.745

Dishion, T.J., McMahon, R.J., 1998. Parental monitoring and the prevention of child and adolescent problem behavior: A conceptual and empirical formulation. Clin. Child Fam. Psychol. Rev. 1, 61–75.

Farmer, R.F., Gau, J.M., Seeley, J.R., Kosty, D.B., Sher, K.J., Lewinsohn, P.M., 2016. Internalizing and externalizing disorders as predictors of alcohol use disorder onset during three developmental periods. Drug Alcohol Depend. 164, 38–46. https://doi.org/10.1016/j.drugalcdep.2016.04.021

Fish, J.N., Russell, B.S., Watson, R.J., Russell, S.T., 2020. Parent-child relationships and sexual minority youth: Implications for adult alcohol abuse. J. Youth Adolesc. 49, 2034–2046. https://doi.org/10.1007/s10964-020-01299-7

Fisher, L.B., Miles, I.W., Austin, S.B., Camargo, C.A., Jr, Colditz, G.A., 2007. Predictors of initiation of alcohol use among us adolescents: Findings from a prospective cohort study. Arch. Pediatr. Adolesc. Med. 161, 959–966. https://doi.org/10.1001/archpedi.161.10.959

Fletcher, A.C., Steinberg, L., Williams-Wheeler, M., 2004. Parental influences on adolescent problem behavior: Revisiting Stattin and Kerr. Child Dev. 75, 781–796. https://doi.org/10.1111/j.1467-8624.2004.00706.x

Forgatch, M.S., Kjøbli, J., 2016. Parent management training-Oregon model: Adapting intervention with rigorous research. Fam. Process 55, 500–513. https://doi.org/10.1111/famp.12224

Fried, A.B., Dunn, M.E., 2012. The Expectancy Challenge Alcohol Literacy Curriculum (ECALC): A single session group intervention to reduce alcohol use. Psychol. Addict. Behav. J. Soc. Psychol. Addict. Behav. 26, 615–620. https://doi.org/10.1037/a0027585

Gioia, G.A., Isquith, P.K., Retzlaff, P.D., Espy, K.A., 2002. Confirmatory factor analysis of the Behavior Rating Inventory of Executive Function (BRIEF) in a clinical sample. Child Neuropsychol. J. Norm. Abnorm. Dev. Child. Adolesc. 8, 249–257. https://doi.org/10.1076/chin.8.4.249.13513

Golub, A., Johnson, B.D., Labouvie, E., 2000. On correcting biases in self-reports of age at first substance use with repeated cross-section analysis. J. Quant. Criminol. 16, 45–68. https://doi.org/10.1023/A:1007573411129

Gosling, S.D., Rentfrow, P.J., Swann, W.B., 2003. A very brief measure of the Big-Five personality domains. J. Res. Personal. 37, 504–528. https://doi.org/10.1016/S0092-6566(03)00046-1

Grant, B.F., Dawson, D.A., 1997. Age at onset of alcohol use and its association with DSM-IV alcohol abuse and dependence: results from the National Longitudinal Alcohol Epidemiologic Survey. J. Subst. Abuse 9, 103–110. https://doi.org/10.1016/s0899-3289(97)90009-2

Gruber, E., DiClemente, R.J., Anderson, M.M., Lodico, M., 1996. Early drinking onset and its association with alcohol use and problem behavior in late adolescence. Prev. Med. 25, 293–300. https://doi.org/10.1006/pmed.1996.0059

Gur, R.C., Richard, J., Hughett, P., Calkins, M.E., Macy, L., Bilker, W.B., Brensinger, C., Gur, R.E., 2010. A cognitive neuroscience-based computerized battery for efficient measurement of individual differences: Standardization and initial construct validation. J. Neurosci. Methods 187, 254–262. https://doi.org/10.1016/j.jneumeth.2009.11.017

Guttmannova, K., Hill, K.G., Bailey, J.A., Lee, J.O., Hartigan, L.A., Hawkins, J.D., Catalano, R.F., 2012. Examining explanatory mechanisms of the effects of early alcohol use on young adult alcohol dependence. J. Stud. Alcohol Drugs 73, 379–390. https://doi.org/10.15288/jsad.2012.73.379

Handren, L.M., Donaldson, C.D., Crano, W.D., 2016. Adolescent alcohol use: Protective and predictive parent, peer, and self-related factors. Prev. Sci. Off. J. Soc. Prev. Res. 17, 862–871. https://doi.org/10.1007/s11121-016-0695-7

Hardee, J.E., Cope, L.M., Martz, M.E., Heitzeg, M.M., 2018. Review of neurobiological influences on externalizing and internalizing pathways to alcohol use disorder. Curr. Behav. Neurosci. Rep. 5, 249–262. https://doi.org/10.1007/s40473-018-0166-5

Hardie, B., 2021. Reconceptualising parental monitoring within a model of goal-directed parental action. New Ideas Psychol. 61, 100847. https://doi.org/10.1016/j.newideapsych.2020.100847

Harrell, F.E., Jr, Califf, R.M., Pryor, D.B., Lee, K.L., Rosati, R.A., 1982. Evaluating the yield of medical tests. JAMA 247, 2543–2546. https://doi.org/10.1001/jama.1982.03320430047030

Harrell, F.E., Lee, K.L., Mark, D.B., 1996. Multivariable prognostic models: issues in developing models, evaluating assumptions and adequacy, and measuring and reducing errors. Stat. Med. 15, 361–387. https://doi.org/10.1002/(SICI)1097-0258(19960229)15:4<361::AID-SIM168>3.0.CO;2-4

Harrell Jr., F.E., 2023. _rms: Regression Modeling Strategies_. R package version 6.7-1.

Hasler, B.P., Graves, J.L., Wallace, M.L., Claudatos, S., Franzen, P.L., Nooner, K.B., Brown, S.A., Tapert, S.F., Baker, F.C., Clark, D.B., 2022. Self-reported sleep and circadian characteristics predict alcohol and cannabis use: A longitudinal analysis of the National Consortium on Alcohol and Neurodevelopment in Adolescence study. Alcohol. Clin. Exp. Res. 46, 848–860. https://doi.org/10.1111/acer.14808

Hasler, B.P., Soehner, A.M., Clark, D.B., 2015. Sleep and circadian contributions to adolescent alcohol use disorder. Alcohol, Special Issue: Sleep, Circadian Rhythms and Alcohol 49, 377–387. https://doi.org/10.1016/j.alcohol.2014.06.010

Hastie, T., Tibshirani, R., Tibshirani, R.J., 2017. Extended comparisons of best subset selection, forward stepwise selection, and the lasso. ArXiv Prepr. ArXiv170708692.

Hastie, T., Tibshirani, Robert, Tibshirani, Ryan, 2020. Best subset, forward stepwise or lasso? Analysis and recommendations based on extensive comparisons. Stat. Sci. 35, 579–592. https://doi.org/10.1214/19-STS733

Hatoum, A.S., Johnson, E.C., Baranger, D.A.A., Paul, S.E., Agrawal, A., Bogdan, R., 2021. Polygenic risk scores for alcohol involvement relate to brain structure in substance-naïve children: Results from the ABCD study. Genes Brain Behav. 20, e12756. https://doi.org/10.1111/gbb.12756

Heikkinen, N., Niskanen, E., Könönen, M., Tolmunen, T., Kekkonen, V., Kivimäki, P., Tanila, H., Laukkanen, E., Vanninen, R., 2017. Alcohol consumption during adolescence is associated with reduced grey matter volumes. Addict. Abingdon Engl. 112, 604–613. https://doi.org/10.1111/add.13697

Hill, S.Y., O’Brien, J., 2015. Psychological and neurobiological precursors of alcohol use disorders in high risk youth. Curr. Addict. Rep. 2, 104–113. https://doi.org/10.1007/s40429-015-0051-1

Hingson, R.W., Heeren, T., Winter, M.R., 2006. Age at drinking onset and alcohol dependence: age at onset, duration, and severity. Arch. Pediatr. Adolesc. Med. 160, 739–746. https://doi.org/10.1001/archpedi.160.7.739

Hingson, R.W., Zha, W., 2009. Age of drinking onset, alcohol use disorders, frequent heavy drinking, and unintentionally injuring oneself and others after drinking. Pediatrics 123, 1477–1484. https://doi.org/10.1542/peds.2008-2176

Honarvar, F., Arfaie, S., Edalati, H., Ghasroddashti, A., Solgi, A., Mashayekhi, M.S., Mofatteh, M., Ren, L.Y., Kwan, A.T.H., Keramatian, K., 2023. Neuroanatomical predictors of problematic alcohol consumption in adolescents: A systematic review of longitudinal studies. Alcohol Alcohol. Oxf. Oxfs. 58, 455–471. https://doi.org/10.1093/alcalc/agad049

Hua, J.P.Y., Piasecki, T.M., McDowell, Y.E., Boness, C.L., Trela, C.J., Merrill, A.M., Sher, K.J., Kerns, J.G., 2020. Alcohol use in young adults associated with cortical gyrification. Drug Alcohol Depend. 209, 107925. https://doi.org/10.1016/j.drugalcdep.2020.107925

Hyatt, C.S., Owens, M.M., Crowe, M.L., Carter, N.T., Lynam, D.R., Miller, J.D., 2020. The quandary of covarying: A brief review and empirical examination of covariate use in structural neuroimaging studies on psychological variables. NeuroImage 205, 116225. https://doi.org/10.1016/j.neuroimage.2019.116225

Infante, M.A., Courtney, K.E., Castro, N., Squeglia, L.M., Jacobus, J., 2018. Adolescent brain surface area pre- and post-cannabis and alcohol initiation. J. Stud. Alcohol Drugs 79, 835–843. https://doi.org/10.15288/jsad.2018.79.835

Jacobus, J., Castro, N., Squeglia, L.M., Meloy, M.J., Brumback, T., Huestis, M.A., Tapert, S.F., 2016. Adolescent cortical thickness pre- and post marijuana and alcohol initiation. Neurotoxicol. Teratol. 57, 20–29. https://doi.org/10.1016/j.ntt.2016.09.005

Kann, L., Olsen, E.O., McManus, T., Harris, W.A., Shanklin, S.L., Flint, K.H., Queen, B., Lowry, R., Chyen, D., Whittle, L., Thornton, J., Lim, C., Yamakawa, Y., Brener, N., Zaza, S., 2016. Sexual Identity, Sex of Sexual Contacts, and Health-Related Behaviors Among Students in Grades 9–12 — United States and Selected Sites, 2015. Morb. Mortal. Wkly. Rep. Surveill. Summ. 65, 1–202. https://doi.org/10.15585/mmwr.ss6509a1

Komro, K.A., Maldonado-Molina, M.M., Tobler, A.L., Bonds, J.R., Muller, K.E., 2007. Effects of home access and availability of alcohol on young adolescents’ alcohol use. Addict. Abingdon Engl. 102, 1597–1608. https://doi.org/10.1111/j.1360-0443.2007.01941.x

Koob, G.F., Volkow, N.D., 2010. Neurocircuitry of addiction. Neuropsychopharmacology 35, 217–238. https://doi.org/10.1038/npp.2009.110

Kühn, S., Mascharek, A., Banaschewski, T., Bodke, A., Bromberg, U., Büchel, C., Quinlan, E.B., Desrivieres, S., Flor, H., Grigis, A., Garavan, H., Gowland, P.A., Heinz, A., Ittermann, B., Martinot, J.-L., Nees, F., Papadopoulos Orfanos, D., Paus, T., Poustka, L., Millenet, S., Fröhner, J.H., Smolka, M.N., Walter, H., Whelan, R., Schumann, G., Lindenberger, U., Gallinat, J., IMAGEN Consortium, 2019. Predicting development of adolescent drinking behaviour from whole brain structure at 14 years of age. eLife 8, e44056. https://doi.org/10.7554/eLife.44056

Kuntsche, E., Rossow, I., Engels, R., Kuntsche, S., 2016. Is “age at first drink” a useful concept in alcohol research and prevention? We doubt that. Addict. Abingdon Engl. 111, 957–965. https://doi.org/10.1111/add.12980

Kuntsche, E., Rossow, I., Simons-Morton, B., Bogt, T.T., Kokkevi, A., Godeau, E., 2013. Not early drinking but early drunkenness is a risk factor for problem behaviors among adolescents from 38 European and North American countries. Alcohol. Clin. Exp. Res. 37, 308–314. https://doi.org/10.1111/j.1530-0277.2012.01895.x

Leung, R.K., Toumbourou, J.W., Hemphill, S.A., 2014. The effect of peer influence and selection processes on adolescent alcohol use: A systematic review of longitudinal studies. Health Psychol. Rev. 8, 426–457. https://doi.org/10.1080/17437199.2011.587961

Loeber, R., Farrington, D.P., Stouthamer-Loeber, M., Van Kammen, W.B., 1998. , in: Antisocial Behavior and Mental Health Problems: Explanatory Factors in Childhood and Adolescence. Psychology Press.

Lynam, D.R., Smith, G.T., Whiteside, S.P., Cyders, M.A., 2006. The UPPS-P: Assessing five personality pathways to impulsive behavior. West Lafayette Purdue Univ. 10.

Maechler, M., Rousseeuw, P., Croux, C., Todorov, V., Ruckstuhl, A., Salibian-Barrera, M., Verbeke, T., Koller, M., Conceicao, E.L.T., di Palma, M.A., 2023. robustbase: basic robust statistics R package version 0.99-0.

Maggs, J.L., Patrick, M.E., Feinstein, L., 2008. Childhood and adolescent predictors of alcohol use and problems in adolescence and adulthood in the National Child Development Study. Addiction 103, 7–22. https://doi.org/10.1111/j.1360-0443.2008.02173.x

Maggs, J.L., Staff, J., Patrick, M.E., Wray-Lake, L., 2019. Very early drinking: Event history models predicting alcohol use initiation from age 4 to 11 years. Addict. Behav. 89, 121–127. https://doi.org/10.1016/j.addbeh.2018.09.030

Manuweera, T., Kisner, M.A., Almira, E., Momenan, R., 2022. Alcohol use disorder-associated structural and functional characteristics of the insula. J. Neurosci. Res. 100, 2077–2089. https://doi.org/10.1002/jnr.25113

Marshal, M.P., Friedman, M.S., Stall, R., King, K.M., Miles, J., Gold, M.A., Bukstein, O.G., Morse, J.Q., 2008. Sexual orientation and adolescent substance use: A meta-analysis and methodological review. Addict. Abingdon Engl. 103, 546–556. https://doi.org/10.1111/j.1360-0443.2008.02149.x

Martin, C.S., Winters, K.C., 1998. Diagnosis and assessment of alcohol use disorders among adolescents. Alcohol Health Res. World 22, 95–105.

Meda, S.A., Hawkins, K.A., Dager, A.D., Tennen, H., Khadka, S., Austad, C.S., Wood, R.M., Raskin, S., Fallahi, C.R., Pearlson, G.D., 2018. Longitudinal effects of alcohol consumption on the hippocampus and parahippocampus in college students. Biol. Psychiatry Cogn. Neurosci. Neuroimaging 3, 610–617. https://doi.org/10.1016/j.bpsc.2018.02.006

Meque, I., Dachew, B.A., Maravilla, J.C., Salom, C., Alati, R., 2019. Externalizing and internalizing symptoms in childhood and adolescence and the risk of alcohol use disorders in young adulthood: A meta-analysis of longitudinal studies. Aust. N. Z. J. Psychiatry 53, 965–975. https://doi.org/10.1177/0004867419844308

Miech, R.A., Johnston, L.D., Patrick, M.E., O’Malley, P.M., Bachman, J.G., Schulenberg, J.E., 2023. Monitoring the Future National Survey results on drug use, 1975-2022: Secondary school students. Inst. Soc. Res.

Mills, R., Mann, M.J., Smith, M.L., Kristjansson, A.L., 2021. Parental support and monitoring as associated with adolescent alcohol and tobacco use by gender and age. BMC Public Health 21, 2000. https://doi.org/10.1186/s12889-021-12119-3

Morean, M.E., Kong, G., Camenga, D.R., Cavallo, D.A., Connell, C., Krishnan-Sarin, S., 2014. First drink to first drunk: Age of onset and delay to intoxication are associated with adolescent alcohol use and binge drinking. Alcohol. Clin. Exp. Res. 38, 2615–2621. https://doi.org/10.1111/acer.12526

Morrison, C.N., Byrnes, H.F., Miller, B.A., Wiehe, S.E., Ponicki, W.R., Wiebe, D.J., 2019. Exposure to alcohol outlets, alcohol access, and alcohol consumption among adolescents. Drug Alcohol Depend. 205, 107622. https://doi.org/10.1016/j.drugalcdep.2019.107622

Nagelkerke, N.J.D., 1991. A note on a general definition of the coefficient of determination. Biometrika 78, 691–692. https://doi.org/10.1093/biomet/78.3.691

National Survey on Drug Use and Health, 2022. National Survey on Drug Use and Health (NSDUH) Population Data [WWW Document]. URL https://www.datafiles.samhsa.gov/dataset/national-survey-drug-use-and-health-2022-nsduh-2022-ds0001 (accessed 6.3.24).

O’Brien, J.W., Hill, S.Y., 2017. Neural predictors of substance use disorders in young adulthood. Psychiatry Res. Neuroimaging 268, 22–26. https://doi.org/10.1016/j.pscychresns.2017.08.006

Petersen, A.C., Crockett, L., Richards, M., Boxer, A., 1988. A self-report measure of pubertal status: Reliability, validity, and initial norms. J. Youth Adolesc. 17, 117–133. https://doi.org/10.1007/BF01537962

Pfefferbaum, A., Kwon, D., Brumback, T., Thomson, W.K., Cummins, K., Tapert, S.F., Brown, S.A., Colrain, I.M., Baker, F.C., Prouty, D., De Bellis, M.D., Clark, D.B., Nagel, B.J., Chu, W., Park, S.H., Pohl, K.M., Sullivan, E.V., 2018. Altered brain developmental trajectories in adolescents after initiating drinking. Am. J. Psychiatry 175, 370–380. https://doi.org/10.1176/appi.ajp.2017.17040469

Pfefferbaum, A., Rohlfing, T., Pohl, K.M., Lane, B., Chu, W., Kwon, D., Nolan Nichols, B., Brown, S.A., Tapert, S.F., Cummins, K., Thompson, W.K., Brumback, T., Meloy, M.J., Jernigan, T.L., Dale, A., Colrain, I.M., Baker, F.C., Prouty, D., De Bellis, M.D., Voyvodic, J.T., Clark, D.B., Luna, B., Chung, T., Nagel, B.J., Sullivan, E.V., 2016. Adolescent development of cortical and white matter structure in the NCANDA sample: Role of sex, ethnicity, puberty, and alcohol drinking. Cereb. Cortex N. Y. N 1991 26, 4101–4121. https://doi.org/10.1093/cercor/bhv205

Pilatti, A., Godoy, J.C., Brussino, S.A., Pautassi, R.M., 2013. Patterns of substance use among Argentinean adolescents and analysis of the effect of age at first alcohol use on substance use behaviors. Addict. Behav. 38, 2847–2850. https://doi.org/10.1016/j.addbeh.2013.08.007

R Core Team, 2023. R: A language and environment for statistical computing.

Rane, R.P., de Man, E.F., Kim, J., Görgen, K., Tschorn, M., Rapp, M.A., Banaschewski, T., Bokde, A.L., Desrivieres, S., Flor, H., Grigis, A., Garavan, H., Gowland, P.A., Brühl, R., Martinot, J.-L., Martinot, M.-L.P., Artiges, E., Nees, F., Papadopoulos Orfanos, D., Lemaitre, H., Paus, T., Poustka, L., Fröhner, J., Robinson, L., Smolka, M.N., Winterer, J., Whelan, R., Schumann, G., Walter, H., Heinz, A., Ritter, K., IMAGEN consortium, 2022. Structural differences in adolescent brains can predict alcohol misuse. eLife 11, e77545. https://doi.org/10.7554/eLife.77545

Rice, J.P., Reich, T., Bucholz, K.K., Neuman, R.J., Fishman, R., Rochberg, N., Hesselbrock, V.M., Nurnberger, J.I., Schuckit, M.A., Begleiter, H., 1995. Comparison of direct interview and family history diagnoses of alcohol dependence. Alcohol. Clin. Exp. Res. 19, 1018–1023. https://doi.org/10.1111/j.1530-0277.1995.tb00983.x

Sartor, C.E., Jackson, K.M., McCutcheon, V.V., Duncan, A.E., Grant, J.D., Werner, K.B., Bucholz, K.K., 2016. Progression from first drink, first intoxication, and regular drinking to alcohol use disorder: A comparison of African American and European American youth. Alcohol. Clin. Exp. Res. 40, 1515–1523. https://doi.org/10.1111/acer.13113

Sartor, C.E., Waldron, M., Duncan, A.E., Grant, J.D., McCutcheon, V.V., Nelson, E.C., Madden, P.A.F., Bucholz, K.K., Heath, A.C., 2013. Childhood sexual abuse and early substance use in adolescent girls: the role of familial influences. Addiction 108, 993–1000. https://doi.org/10.1111/add.12115

Schober, P., Vetter, T.R., 2018. Survival analysis and interpretation of time-to-event data: The tortoise and the hare. Anesth. Analg. 127, 792–798. https://doi.org/10.1213/ANE.0000000000003653

Sellers, C.M., McManama O’Brien, K.H., Hernandez, L., Spirito, A., 2018. Adolescent alcohol use: The effects of parental knowledge, peer substance use, and peer tolerance of use. J. Soc. Soc. Work Res. 9, 69–87. https://doi.org/10.1086/695809

Smith, C.L., Cooper, B.R., Miguel, A., Hill, L., Roll, J., McPherson, S., 2021. Predictors of cannabis and tobacco co-use in youth: exploring the mediating role of age at first use in the population assessment of tobacco health (PATH) study. J. Cannabis Res. 3, 16. https://doi.org/10.1186/s42238-021-00072-2

Spilsbury, J.C., Drotar, D., Rosen, C.L., Redline, S., 2007. The Cleveland Adolescent Sleepiness Questionnaire. J. Clin. Sleep Med. JCSM Off. Publ. Am. Acad. Sleep Med. 3, 603–612.

Squeglia, L.M., Ball, T.M., Jacobus, J., Brumback, T., McKenna, B.S., Nguyen-Louie, T.T., Sorg, S.F., Paulus, M.P., Tapert, S.F., 2017. Neural predictors of initiating alcohol use during adolescence. Am. J. Psychiatry 174, 172–185. https://doi.org/10.1176/appi.ajp.2016.15121587

Squeglia, L.M., Cservenka, A., 2017. Adolescence and drug use vulnerability: Findings from neuroimaging. Curr. Opin. Behav. Sci. 13, 164–170. https://doi.org/10.1016/j.cobeha.2016.12.005

Squeglia, L.M., Rinker, D.A., Bartsch, H., Castro, N., Chung, Y., Dale, A.M., Jernigan, T.L., Tapert, S.F., 2014. Brain volume reductions in adolescent heavy drinkers. Dev. Cogn. Neurosci. 9, 117–125. https://doi.org/10.1016/j.dcn.2014.02.005

Tobler, A.L., Komro, K.A., Maldonado-Molina, M.M., 2009. Relationship between neighborhood context, family management practices and alcohol use among urban, multi-ethnic, young adolescents. Prev. Sci. Off. J. Soc. Prev. Res. 10, 313–324. https://doi.org/10.1007/s11121-009-0133-1

Trucco, E.M., 2020. A review of psychosocial factors linked to adolescent substance use. Pharmacol. Biochem. Behav. 196, 172969. https://doi.org/10.1016/j.pbb.2020.172969

Trujillo, C.A., Obando, D., Trujillo, A., 2019. An examination of the association between early initiation of substance use and interrelated multilevel risk and protective factors among adolescents. PLOS ONE 14, e0225384. https://doi.org/10.1371/journal.pone.0225384

Uddin, L.Q., Nomi, J.S., Hebert-Seropian, B., Ghaziri, J., Boucher, O., 2017. Structure and function of the human insula. J. Clin. Neurophysiol. Off. Publ. Am. Electroencephalogr. Soc. 34, 300–306. https://doi.org/10.1097/WNP.0000000000000377

Urošević, S., Collins, P., Muetzel, R., Schissel, A., Lim, K.O., Luciana, M., 2015. Effects of reward sensitivity and regional brain volumes on substance use initiation in adolescence. Soc. Cogn. Affect. Neurosci. 10, 106–113. https://doi.org/10.1093/scan/nsu022

U.S. Census Bureau, 2023a. County Business Patterns Datasets [WWW Document]. URL https://www.census.gov/programs-surveys/cbp/data/datasets.html (accessed 12.20.23).

U.S. Census Bureau, 2023b. American Community Survey (ACS) [WWW Document]. Census.gov. URL https://www.census.gov/programs-surveys/acs (accessed 12.20.23).

U.S. Census Bureau, 2023c. Census Glossary [WWW Document]. Glossary. URL https://www.census.gov/glossary/?term=Unemployed (accessed 12.20.23).

U.S. Census Bureau, 2022. American Community Survey and Puerto Rico Community Survey Design and Methodology. American Community Survey (ACS).

Visser, L., de Winter, A.F., Vollebergh, W.A.M., Verhulst, F.C., Reijneveld, S.A., 2015. Do child’s psychosocial functioning, and parent and family characteristics predict early alcohol use? The TRAILS Study. Eur. J. Public Health 25, 38–43. https://doi.org/10.1093/eurpub/cku072

Warner, L.A., White, H.R., 2003. Longitudinal effects of age at onset and first drinking situations on problem drinking. Subst. Use Misuse 38, 1983–2016. https://doi.org/10.1081/JA-120025123

Whelan, R., Watts, R., Orr, C.A., Althoff, R.R., Artiges, E., Banaschewski, T., Barker, G.J., Bokde, A.L.W., Büchel, C., Carvalho, F.M., Conrod, P.J., Flor, H., Fauth-Bühler, M., Frouin, V., Gallinat, J., Gan, G., Gowland, P., Heinz, A., Ittermann, B., Lawrence, C., Mann, K., Martinot, J.-L., Nees, F., Ortiz, N., Paillère-Martinot, M.-L., Paus, T., Pausova, Z., Rietschel, M., Robbins, T.W., Smolka, M.N., Ströhle, A., Schumann, G., Garavan, H., 2014. Neuropsychosocial profiles of current and future adolescent alcohol misusers. Nature 512, 185–189. https://doi.org/10.1038/nature13402

Zeileis, A., Kleiber, C., Jackman, S., 2008. Regression Models for Count Data in R. J. Stat. Softw. 27.
